# Ube2v1-mediated ubiquitination and degradation of Sirt1 promotes metastasis of colorectal cancer by epigenetically suppressing autophagy

**DOI:** 10.1186/s13045-018-0638-9

**Published:** 2018-07-17

**Authors:** Tong Shen, Ling-Dong Cai, Yu-Hong Liu, Shi Li, Wen-Juan Gan, Xiu-Ming Li, Jing-Ru Wang, Peng-Da Guo, Qun Zhou, Xing-Xing Lu, Li-Na Sun, Jian-Ming Li

**Affiliations:** 1 Department of Pathology, Soochow University Medical School, Suzhou, 215123 People’s Republic of China; 2 Department of Pathology, Baoan Hospital, Southern Medical University, Shenzhen, 518101 People’s Republic of China

**Keywords:** Ubiquitin-conjugating E2 enzyme, Autophagy, Epithelial mesenchymal transition, Metastasis, Colorectal cancer

## Abstract

**Background:**

Ubiquitination is a basic post-translational modification for cellular homeostasis, and members of the conjugating enzyme (E2) family are the key components of the ubiquitin–proteasome system. However, the role of E2 family in colorectal cancer (CRC) is largely unknown. Our study aimed to investigate the role of Ube2v1, one of the ubiquitin-conjugating E2 enzyme variant proteins (Ube2v) but without the conserved cysteine residue required for the catalytic activity of E2s, in CRC.

**Methods:**

Immunohistochemistry and real-time RT-PCR were used to study the expressions of Ube2v1 at protein and mRNA levels in CRC, respectively. Western blotting and immunofluorescence, transmission electron microscopy, and in vivo rescue experiments were used to study the functional effects of Ube2v1 on autophagy and EMT program. Quantitative mass spectrometry, immunoprecipitation, ubiquitination assay, western blotting, and real-time RT-PCR were used to analyze the effects of Ube2v1 on histone H4 lysine 16 acetylation, interaction with Sirt1, ubiquitination of Sirt1, and autophagy-related gene expression.

**Results:**

Ube2v1 was elevated in CRC samples, and its increased expression was correlated with poorer survival of CRC patients. Ube2v1 promoted migration and invasion of CRC cells in vitro and tumor growth and metastasis of CRC cells in vivo. Interestingly, Ube2v1suppressed autophagy program and promoted epithelial mesenchymal transition (EMT) and metastasis of CRC cells in an autophagy-dependent pattern in vitro and in vivo. Moreover, both rapamycin and trehalose attenuated the enhanced Ube2v1-mediated lung metastasis by inducing the autophagy pathway in an orthotropic mouse xenograft model of lung metastasis. Mechanistically, Ube2v1 promoted Ubc13-mediated ubiquitination and degradation of Sirt1 and inhibited histone H4 lysine 16 acetylation, and finally epigenetically suppressed autophagy gene expression in CRC.

**Conclusions:**

Our study functionally links Ube2v1, an E2 member in the ubiquitin–proteasome system, to autophagy program, thereby shedding light on developing Ube2v1 targeted therapy for CRC patients.

**Electronic supplementary material:**

The online version of this article (10.1186/s13045-018-0638-9) contains supplementary material, which is available to authorized users.

## Background

Ubiquitination mediated by the ubiquitin–proteasome system is basically required for the protein homeostasis in the cells [1]. The conjugation of ubiquitin to substrates usually involves three key steps, an activation step initiated by E1, an intermediate step covalently linking ubiquitin to a conjugating enzyme (E2), and a final step usually facilitated by a ligase enzyme (E3) which catalyzes the transfer of ubiquitin from the E2 to the protein substrate [2]. Members of the E2 family are key components for the ubiquitin–proteasome system. However, the role of the E2 family in autophagy and colorectal cancer (CRC) progression remains poorly defined.

Autophagy is an alternative mechanism to maintain cellular homeostasis, which is characterized by an autophagosome-dependent lysosomal degradation of long-lived proteins and unneeded organelles [3]. Although autophagy and proteasomal mediated lysosomal degradation use distinct components, they may also have some shared specific mechanisms [4–7]. Recently, the roles of autophagy in malignant transformation and cancer progression are concerned [8–18]. However, the roles of autophagy in tumorigenesis and progression are still not well characterized.

Ubiquitin-conjugating E2 enzyme variant proteins constitute a distinct subfamily within the E2 protein family, which lack the conserved cysteine residue needed for the catalytic activity of E2s [19, 20]. The functions of ubiquitin-conjugating E2 enzyme variant proteins are not well understood. Ubiquitin-conjugating E2 enzyme variant 1 (Ube2v1), one member of ubiquitin-conjugating E2 enzyme variant proteins, has been controversially suggested as both a candidate oncogene or a tumor suppressor [19, 21–23]. In addition, Ube2v1 has been found as one of the key components of TRAF6 to control NF-κB activation [20, 24–28]. Nevertheless, the role of Ube2v1 in autophagy and cancer including CRC and the mechanisms involved are still largely unknown.

Here, we reported that Ube2v1 promoted ubiquitination and degradation of Sirt1 by the help of Ubc13, inhibited histone H4 lysine 16 acetylation, and finally epigenetically suppressed gene expression of autophagy genes. Importantly, Ube2v1 promoted epithelial mesenchymal transition (EMT) and metastasis using an autophagy-related mechanism. Ube2v1 is employed as a new effective therapeutic target for CRC.

## Methods

### Cell lines and reagents

The human CRC cell lines DLD-1, SW480, and HCT116 (Additional file 1) were obtained from the Cell Bank at the Chinese Academy of Sciences (Shanghai, People’s Republic of China) and cultured in RPMI-1640 or DMEM containing 10% fetal bovine serum. Human Ube2v1 cDNA were cloned into the GV218 expression vector (Genechem) and GV248 expression vector (Genechem), respectively. Stable overexpression and low expression Ube2v1 cell lines were selected by puromycin (1 mg/ml) and confirmed by RT-qPCR and western blots. Cells were transfected using the Lipofectamine 2000 (Invitrogen, CA, USA). siRNAs (RiboBio silencer siRNA: negative control, Ube2v1 [ID#siG1463152832 and siG1463152844]), Ubc13 (ID#stB000527A and ID#stB0005287B), Sirt1 (ID#siB09917110134 and ID#siB09917110218), ATG5 (ID#siB07103014081), ATG7 (ID#siB101210103531) were transfected at a final concentration of 10 nM using Lipofectamine™RNAiMAX (Invitrogen). 3-MA (SigmaAldrich) was dissolved in DMSO (50 mM stock solution) and always used at a final concentration of 5 mM unless otherwise indicated. BafA1 was diluted in dimethylsulfoxide (DMSO) and used at a final working concentration of 1 μmol/l for 6 and 12 h, respectively. NSC697923 (Selleckchem), specifically inhibiting the activity of Ubc13-Uev1A, was dissolved in DMSO and used at 0.5 and 1 μM for 5 min. The proteasome inhibitor MG132 (Sigma Aldrich) was dissolved in DMSO and added in media at a final concentration of 10 μM for 4 h. Cycloheximide (CHX, Cell Signaling Technology) was added in the culture medium at concentrations of 100 μg/ml for 3, 6, and 9 h, respectively. Sirt1 expression construct has been described previously [29]. The constructs for GFP-LC3, pCMV-myc-Ube2v1, GFP-Ubc13, pCMV-myc-Ubc13, hemagglutinin (HA)-tagged ubiquitin gene (HA-Ub), and mCherry-GFP-LC3B were generated by PCR and confirmed by sequencing.

### Lung metastases in vivo

To analyze lung metastases, CRC cells (1 × 10^7^/0.2 ml) were injected into the lateral tail vein of 6-week-old BALB/C-nu mice. Ten weeks after tail vein injection, mice were sacrificed, and the lungs were weighed. The lung metastases were examined using H&E staining. The number of surface metastases per lung was determined under a dissecting microscope. For comparison, we also prepared rapamycin for IP injection. Rapamycin (Tokyo Chemical Industry) was dissolved in ethanol, which was then diluted with PBS to a final concentration of 1 mg/ml directly before use. Mice were administered daily via intraperitoneal injections of 5 mg/kg rapamycin for 3 days per week following injection of SW480/Ube2v1 into the lateral tail vein for 2 weeks. Lung pairs were prepared for immunohistochemistry. All animal protocols were performed with the approval of the ethics committee of the Soochow University.

### The wound-healing assay

The cancer cells were cultured in 6-well plates and grown in medium containing 10% FBS to nearly confluent cell monolayer, then carefully scratched using a plastic pipette tip to draw a linear “wound” in the cell monolayer of each well. The monolayer was washed twice with PBS to remove debris or the detached cells from the monolayer. The cells were incubated at 37 °C and monitored by time lapse (photographed per 20 min for 12 h) in the Nikon microscope Ti-S (Japan). Under the microscope, the number of cells that migrated into the cell-free zone, base on the zero line of the linear “wound,” was evaluated. The experiments were performed thrice in triplicate and were counted double blind by at least two investigators.

### Transwell migration assay

For transwell migration assays, 5 × 10^5^ cells were plated in the top chamber (serum-free medium) onto the non-coated membrane (24-well insert; pore size, 8 μm; Corning Costar) and allowed to migrate toward 10% serum-containing medium in the lower chamber. Cells were fixed after 36 h of incubation with methanol and stained with Giemsa solution. The number of cells invading through the membrane was counted under a light microscope (× 40, three random fields per well).

### Transwell invasion assay

For invasion assay, 5 × 10^5^ cells were plated in the top chamber onto the Matrigel-coated membrane (24-well insert; pore size, 8 μm; Corning Costar). Each well was coated freshly with Matrigel (60 μg; BD Bioscience) before the invasion assay. Cells were plated in medium without serum or growth factors, and medium supplemented with serum was used as a chemoattractant in the lower chamber. The cells were incubated for 48 h, and cells that did invade through the pores were removed by a cotton swab. Cells on the lower surface of the membrane were fixed with methanol and stained with Giemsa solution. The number of cells invading through the membrane was counted under a light microscope (× 40, three random fields per well).

### Human CRC samples

Surgically resected CRC specimens with paired normal mucosal counterparts were obtained from The First Affiliated Hospital of Soochow University. All procedures involving human tumor biopsies were performed with the approval of the ethics committee of the Soochow University. The patients had given written informed consent.

### RNA isolation and qPCR

Total RNA was isolated using the Trizol (Invitrogen) according to the manufacturer’s instructions. For mRNA, cDNA was generated from 1 μg total RNA per sample using the Transcriptor First Strand cDNA Synthesis Kit (Roche). qPCR was performed by using the ABI StepOne Plus and the SYBR® Select Master Mix (ABI). mRNA expression was normalized using detection of 18S ribosomal RNA. Results are represented as fold induction using the ΔΔCt method with the control set to 1. The sequences of qPCR primers are listed in Additional file 2: Table S1.

### Western blot analysis and antibodies

Cell lysates were collected in RIPA lysis buffer (1% Triton-X-100, 20 mM Tris, pH 7.5, 137 mM NaCl, 1 mM EGTA, 10% glycerol, 1.5 mM MgCl_2_, and protease inhibitor mixture and phosphatase inhibitors; latter 2 were from Roche). Lysates were sonicated and centrifuged at 4 °C. Per lane, whole-cell lysate was separated on 12% SDS-acrylamide gels and transferred on Immobilon PVDF membranes (Millipore). The membranes were probed with primary antibodies overnight at 4 °C and incubated for 1 h with secondary peroxidase-conjugated antibodies (CST). Chemiluminescent signals were then developed with Lumiglo reagent (Cell Signaling Technology) and detected by the ChemiDoc XRS gel documentation system (Bio-rad). Antibodies include anti-Ube2v1 (Abcam, monoclonal ab151725), anti-E-cadherin (Santa Cruz, monoclonal sc-21791), anti-N-cadherin (Boster, polyclonal BA0673), anti-Fibronectin (Boster, polyclonal BA1771), anti-Vimentin (Abcam, monoclonal, ab8978), anti-β-Catenin (Cell Signaling Technology, monoclonal #8480), anti-Twist1 (Proteintech, 25465), anti-Snai1 (Bioss, bs-2441R), anti-LC3B (CST, monoclonal 3868), anti-SQSTM1/p62 (CST, polyclonal 5114), anti-H4K16ac (Immunoway, polyclonal YM3317), anti-Beclin1 (Boster, polyclonal PB0014), anti-histone H3 (Abcam, polyclonal ab1791), and anti-Sirt1 (Santa Cruz, monoclonal sc-74504).

### Co-immunoprecipitation and ubiquitination assays

Antibodies include anti-Sirt1 (Santa Cruz, polyclonal sc-15404), anti-HA-probe (Santa Cruz, polyclonal sc-805), anti-Myc (Proteintech Group, Inc., 10828-1-AP), anti-GFP (GeneTex, Inc., Monoclonal GT859), anti-Ubc13 (Cell Signaling Technology, Monoclonal #6999), and anti-IgG (Santa Cruz, polyclonal sc-66931). SW480 cells were lysed in Tris/HCl, pH 7.5, buffered with 1% Triton containing protease inhibitors as described above. Supernatant was incubated with appropriate antibody (2 μg) for at least 90 min at 4 °C followed by incubation overnight with Protein A/G-Sepharose beads (GE Healthcare). After overnight incubation, the agarose beads were washed four times with cold lysis buffer, incubated for 10 min at 108 °C with loading buffer, and subjected to SDS-PAGE and western blot analysis. To detect Sirt1 ubiquitination, 10 mM *N*-ethylmaleimide was included in the lysis buffer containing a protease inhibitor cocktail (Roche, Hongkong, China).

### Immunohistochemistry

Paraffin-embedded slides were incubated with primary antibodies: anti-Ube2v1 (Abcam, polyclonal ab88679), anti-E-cadherin (Dako, monoclonal M3612), anti-Fibronectin (Boster, polyclonal BA1771), anti-Vimentin (Abcam, monoclonal, ab8978), anti-β-catenin (Cell Signaling Technology, monoclonal #8480), anti-SQSTM1/p62 (CST, polyclonal 5114), and anti-Beclin1 (Boster, polyclonal PB0014). Staining was done on a SPlink Detection Kit (SP-9000). We quantified staining intensity and percentage of stained cells. Positive tumor cells were quantified by two independent observers. The staining intensity was scored on a scale of 0–3 as negative (0), weak (1), medium (2), or strong (3). The extent of the staining, defined as the percentage of positive staining areas of tumor cells in relation to the whole tumor area, was scored on a scale of 0 (0%), 1 (1–25%), 2 (26–50%), 3 (51–75%), and 4 (76–100%). An overall protein expression score (overall score range, 0–12) was calculated by multiplying the intensity and extent positively scores.

### Immunofluorescence microscopy

Cells were permeabilized with 0.3% Triton X-100 for 10 min followed by fixation with 2–4% Methanal for 15 min, and blocked with 3% sheep serum at room temperature for 60 min. Then, probed with primary antibodies anti-LC3B, anti-E-cadherin, anti-β-catenin, anti-Vimentine, and anti-SQSTM1/p62 were described before overnight at room temperature, and cells were washed three times with PBS. Stained with anti-rabbit IgG H&L (FITC) (abcam #ab6717) and anti-mouse IgG H&L (FITC) (abcam #ab6785) for 1 h at room temperature, and then the cells were washed three times with PBS. SW480 cells expressing GFP-RFP-LC3 were treated with Torin (Cayman Chemical) 250 nM for 6 h. Nuclei were visualized by staining with DAPI (Sigma Aldrich, USA) for 2 min. The stained cells were observed with an inverted fluorescence microscope (Nikon Ni-U). Autophagy was measured by quantitation of GFP-LC3 puncta per cell using fluorescence microscopy. All GFP-LC3 puncta quantitation was performed by an observer blinded to experimental condition.

### Electron microscopy

Cells plated at 2 × 10^6^ cells/mL were treated. In a primary case, each sample was fixed with 2% glutaraldehyde–paraformaldehyde in 0.1 M phosphate buffer (PB), pH 7.4 for 2 h and washed three times for 30 min each in 0.1 M PB. Samples were then postfixed with 1% OsO4 dissolved in 0.1 M PB for 2 h and dehydrated in an ascending gradual series (50–100%) of ethanol and infiltrated with propylene oxide. After sectioning and staining with uranyl acetate and lead citrate, they were observed under an electron microscope (JEM 1200EX; JEOL).

### Quantitative mass spectrometry

Proteins were extracted from SW480cell lines with stable overexpressioning Ube2v1 and its control. Total protein concentrations were estimated with the bicinchoninic acid assay (Pierce BCA Protein Assay Kit; Thermo Fisher Scientific Inc. #23227, Waltham, MA, USA). A quantity of 50 mg of protein from each sample was used following the manufacturer’s protocol (Expedeon, San Diego, CA, USA) with a minor modification by substituting urea with triethylammonium bicarbonate (TEAB) buffer for sample washes to avoid the primary amine group containing chemical that would interfere with TMT labeling. Each sample was digested with sequencing-grade trypsin (Promega, Fitchburg, WI, USA) in 500 mM TEAB buffer overnight in an enzyme to substrate ratio of 1:100 (wt:wt) at room temperature with gentle shaking, followed by a second digestion for 4 h with the same amount of trypsin. The digested peptide from different samples were labeled with tandem mass tags (TMT) reagents (Thermo, Pierce Biotechnology) according to the manufacturer’s instruction (TMT 127, 126 for the samples). Briefly, the TMT label reagents were dissolved by anhydrous acetonitrile and carefully added to each digestion products. The reaction was performed for 1 h at room temperature, and hydroxylamine was used to quench the reaction. The TMT-labeled peptides were desalted using the stage tips. For LC-MS/MS analysis, the MS/MS spectra from each LC-MS/MS run were searched against the selected database using an in-house Mascot or Proteome Discovery searching algorithm. Peptides that have 127/126 scores > 1.2 and 126/127 scores < 0.8 were used for protein identification, and MS/MS spectra for all matched peptides were manually interpreted and confirmed. The QMS experiments were repeated for three times, and similar results were obtained.

### Statistical analysis

Data were expressed as mean ± SD. Each experiment was performed in at least three repetitions. Student’s *t* test (unpaired, two-tailed) was used to compare two groups of independent samples. One-way ANOVA was used for multiple comparisons. For analyses of associations of Ube2v1 expression with clinical parameters of CRC patients, the chi-square test was performed. *P* values of 0.05 or less were considered statistically significant.

## Results

### Ube2v1 suppresses autophagy in colorectal cancer

**Fig. 1:**
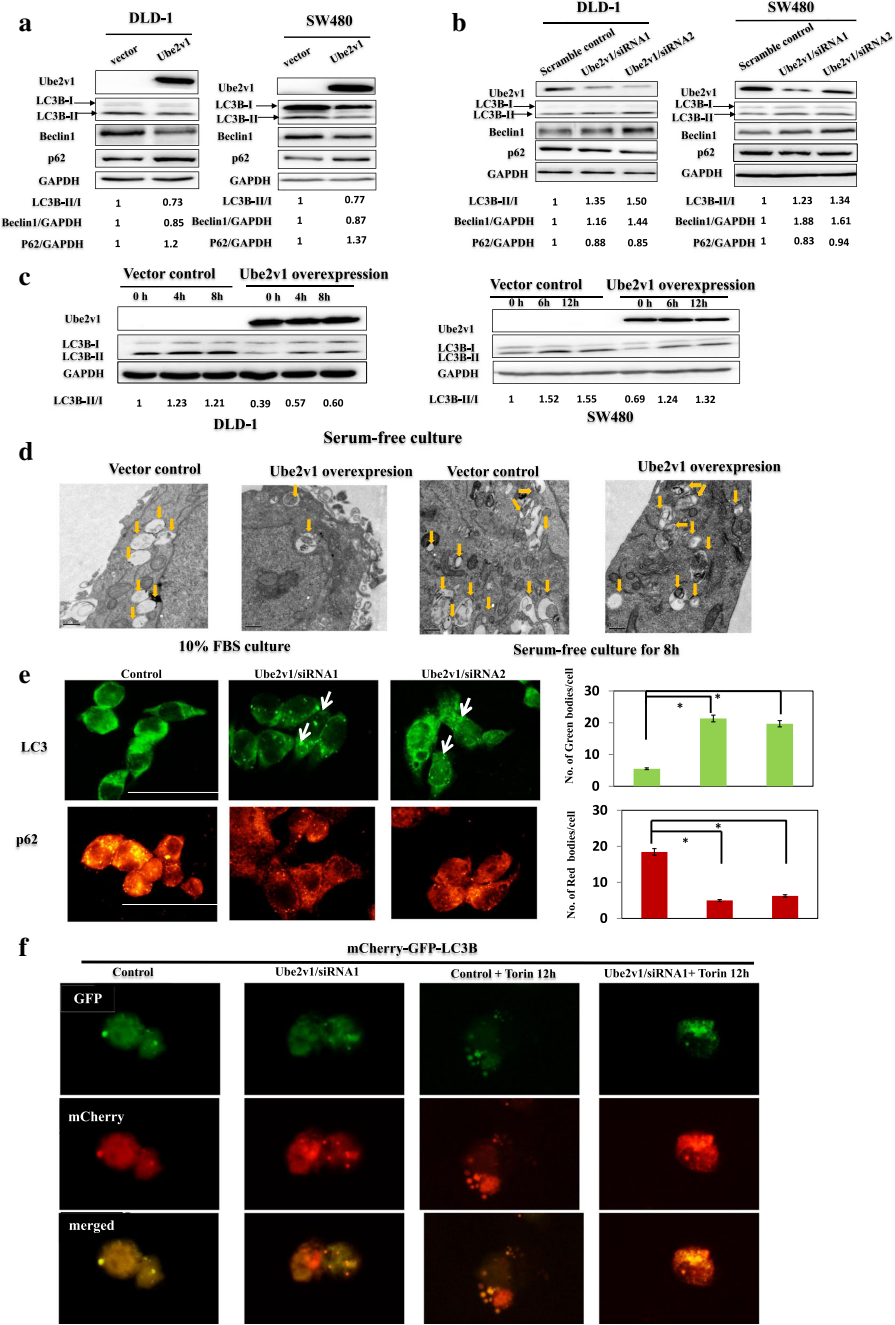
The effects of Ube2v1 on autophagy program in colorectal cancer. **a**, **b** Protein expressions of LC3-II, Beclin1, and P62 level were examined by western blots when Ube2v1was overexpressed (**a**) or knocked down (**b**) in DLD-1 and SW480 cells under normal medium culture condition. **c** Protein expression of LC3-II was examined by western blots when Ube2v1was overexpressed in DLD-1 and SW480 cells under starvation in Hank’s buffered saline solution (HBSS). **d** Enlarged multivesicular structures corresponding to autophagic vacuole in cells with stable Ube2v1 overexpression under normal condition or starvation condition were observed by transmission electron microscope (TEM). **e** Immunofluorescence staining of endogenous LC3 or P62 puncta was analyzed in SW480 cells after Ube2v1 expression was knocked down. **f** A mCherry-GFP-LC3B reporter was transfected into the cells to monitor the autophagy progression from the autophagosome characterized with green fluorescent protein (GFP) to autolysosome characterized with red fluorescent protein (RFP) after Ube2v1 knockdown or and Torin (concentration) treatment for 12 h

The role of autophagy in cancer progression is recently suggested [18]. However, the role of E2 family in autophagy is largely unclear. In our experiments, changes of LC3-II, Beclin1, and p62 protein levels were used as indicators of autophagy program. Surprisingly, decreased LC3-II and Beclin1 levels and increased P62 expression were observed when Ube2v1was overexpressed in DLD-1 and SW480 cells (Fig. 1a). Moreover, Ube2v1 knockdown in DLD-1 and SW480 cells led to increased LC3-II and Beclin1 levels and decreased P62 level (Fig. 1b). Given that starvation will initiate the autophagy program, we further evaluated the effects of Ube2v1 on starvation-mediated autophagy program. Cells were cultured under starvation in Hank’s buffered saline solution (HBSS) for different interval, and we found that Ube2v1 overexpression attenuated the starvation initiated autophagy program (Fig. 1c). Moreover, even when the cells were treated with bafilomycin A1 (BafA1), an autophagy inhibitor which blocks autophagosome–lysosome fusion and leads to accumulation of autophosome characterized with increased expression of LC3-II, Ube2v1 overexpression still had suppressive effect on autophagy under both normal culture and starvation conditions (Additional file 3: Figure S1). Ultrastructurally, the number of enlarged multivesicular structures corresponding to autophagic vacuoles was significantly decreased in SW480 cells with stable Ube2v1 overexpression under both normal condition or starvation condition by transmission electron microscope (TEM) and (Fig. 1d). To further quantify the changes of autophagy flux affected by Ube2v1, immunofluorescence staining of endogenous LC3 or P62 puncta were analyzed in SW480 cells after Ube2v1 expression was knocked down. Our results showed that Ube2v1 knockdown increased autophagy flux in SW480 cells (Fig. 1e). To further study the role of Ube2v1 on autophagy flux, A mCherry-GFP-LC3 assay were used to monitor the autophagy progression from the autophagosome labeled with green fluorescent protein (GFP) to autolysosome labeled with red fluorescent protein (RFP). We found that Ube2v1 knockdown led to increased autophagy flux, indicating as increasing expression of both green and red puncta within the cells (Fig. 1f). Torin, an inhibitor for mTOR pathway, was used to induce the autophagy program as a positive control in our experiment. Interestingly, Ube2v1 knockdown and Torin treatment had synergistic effect on autophagy (Fig. 1f). Together, these data demonstrate that Ube2v1 suppresses the autophagy program in CRC cells.

### Ube2v1 inhibits histone H4 lysine 16 acetylation by downregulating expression of Sirt1 and epigenetically suppresses gene expression of autophagy-related genes in CRC

**Fig. 2:**
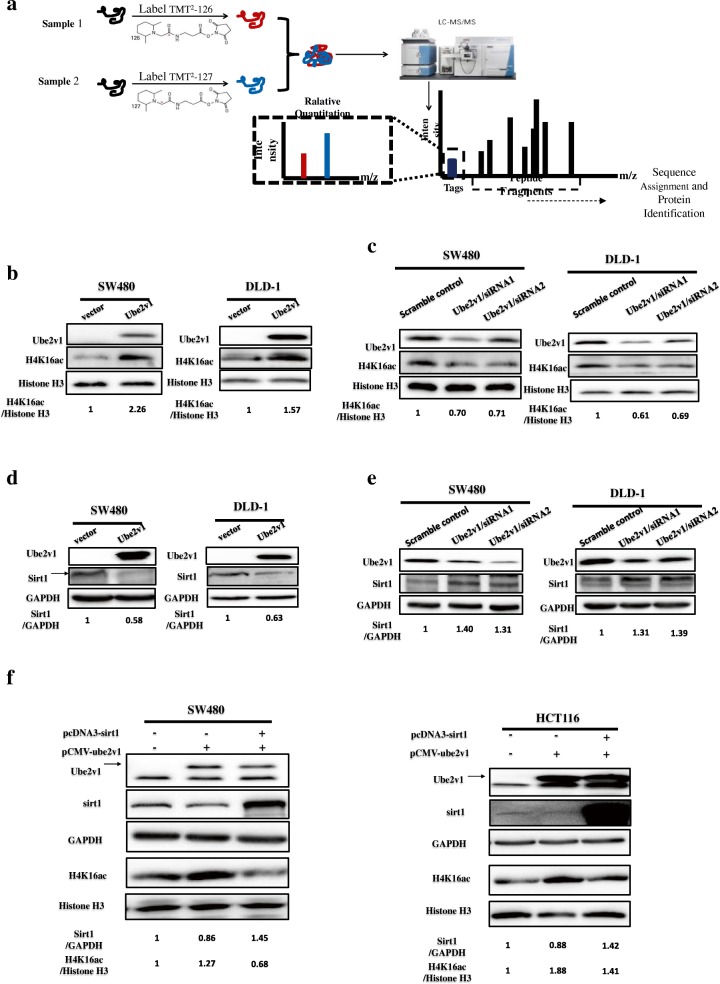
The effects of Ube2v1 on histone H4 lysine 16 acetylation and expression of Sirt1. **a** Quantitative mass spectrometry was used to analyze differential proteins between control cells and cells with Ube2v1 stable overexpression. **b**, **c** Expression of histone H4 lysine 16 acetylation (H4K16ac) in histone extracts was examined after Ube2v1 was overexpressed (**b**) or knocked down (**c**) in SW480 and DLD-1 cells. **d**, **e** Sirt1 expression was detected after Ube2v1 was overexpressed (**d**) or knocked down (**e**) in SW480 and DLD-1 cells. **f** The effects of Ube2v1 overexpression on H4K16ac with or without Sirt1 overexpression

To delineate how Ube2v1 interferes with autophagy program, quantitative mass spectrometry was used to analyze differentially expressed proteins between control cells and cells with Ube2v1 stable overexpression (Fig. 2a). Interestingly, histones were enriched in these differentially expressed proteins (Additional file 2: Table S2), suggesting Ube2v1 may play a role in chromatin modification. It was suggested that reduction of histone H4 lysine 16 acetylation (H4K16ac) could lead to induction of autophagy [30, 31]. Based on our results from quantitative mass spectrometry, we speculated that histone H4 lysine 16 acetylation (H4K16ac) might be affected by Ube2v1. Surprisingly, we observed that H4K16acin histone extracts was significantly increased in Ube2v1 overexpressed SW480 and DLD-1 cells (Fig. 2b). Accordingly, H4K16ac in histone extracts was significantly decreased after Ube2v1 was knocked down in SW480 and DLD-1 cells (Fig. 2c). As we know, H4K16ac is one of the primary histone targets of Sirtuin 1 (Sirt1) [32]. Therefore, we postulated that the regulatory effects of Ube2v1 on H4K16ac are related to the effects of Ube2v1 on Sirt1. Interestingly, we found that Sirt1 expression was significantly decreased in Ube2v1 overexpressed SW480 and DLD-1 cells (Fig. 2d), while Sirt1 expression was significantly increased after Ube2v1was knocked down in SW480 and DLD-1 cells (Fig. 2e). Moreover, the suppressive effects of Ube2v1 overexpression on H4K16ac can be successfully rescued by Sirt1 overexpression (Fig. 2f), suggesting that the role of Ube2v1 in autophagy program is at least partly depending on Sirt1 function.

Sirt1 is a key epigenetic player for gene regulation of autophagy genes. Thus, the regulatory effects of Ube2v1 on Sirt1 and H4K16ac might involve the epigenetic suppression of autophagy gene expression in CRC. qRT-PCR analysis showed that mRNA levels of many autophagy-related genes (LC3, Beclin1, ATG16L1, ATG3, ATG5, ATG7, ATG12, ATG10, ATG4a, ATG4b, ATG4c, and ATG4d) were significantly elevated when Ube2v1 expression was knocked down in SW480 cells (Additional file 3: Figure S2A), which were similar to gene expression patterns of autophagy genes induced by Sirt1 overexpression (Additional file 3: Figure S2D). In con7trast, Ube2v1 overexpression in SW480 cells significantly inhibited autophagy gene expression (Additional file 3: Figure S2B), which were similar to those induced by Sirt1 knockdown (Additional file 3: Figure S2C).

Generally, our study shows Ube2v1 as an epigenetic regulator for autophagy gene expression.

### Ube2v1 destabilizes Sirt1 and promotes Sirt1 ubiquitination by the help of Ubc13

To determine the mechanism by which Ube2v1 regulates Sirt1 expression, we examined whether Ube2v1 affects the turnover of Sirt1 protein. CRC cells were treated with cycloheximide (CHX), an inhibitor of protein synthesis, and Sirt1 expression was determined by western blotting at various intervals. We found that silencing of Ube2v1 in SW480 cells significantly increased the half-life of Sirt1 (Additional file 3: Figure S3A), while overexpression of Ube2v1 in SW480 cells significantly decreased the half-life of Sirt1 (Additional file 3: Figure S3B), indicating that Ube2v1 promotes the turnover of Sirt1 protein.

Furthermore, we determine whether Ube2v1 affects Sirt1 ubiquitination. Ube2v1 overexpressed SW480 cells were treated with MG132 to block the degradation of Sirt1. Interestingly, overexpression of Ube2v1 significantly increased the ubiquitination of Sirt1 (Additional file 3: Figure S3C). Consistent with this finding, knockdown of Ube2v1 expression led to the dramatic decrease of Sirt1 ubiquitination in the presence or absence of MG132 (Additional file 3: Figure S3D).

**Fig. 3:**
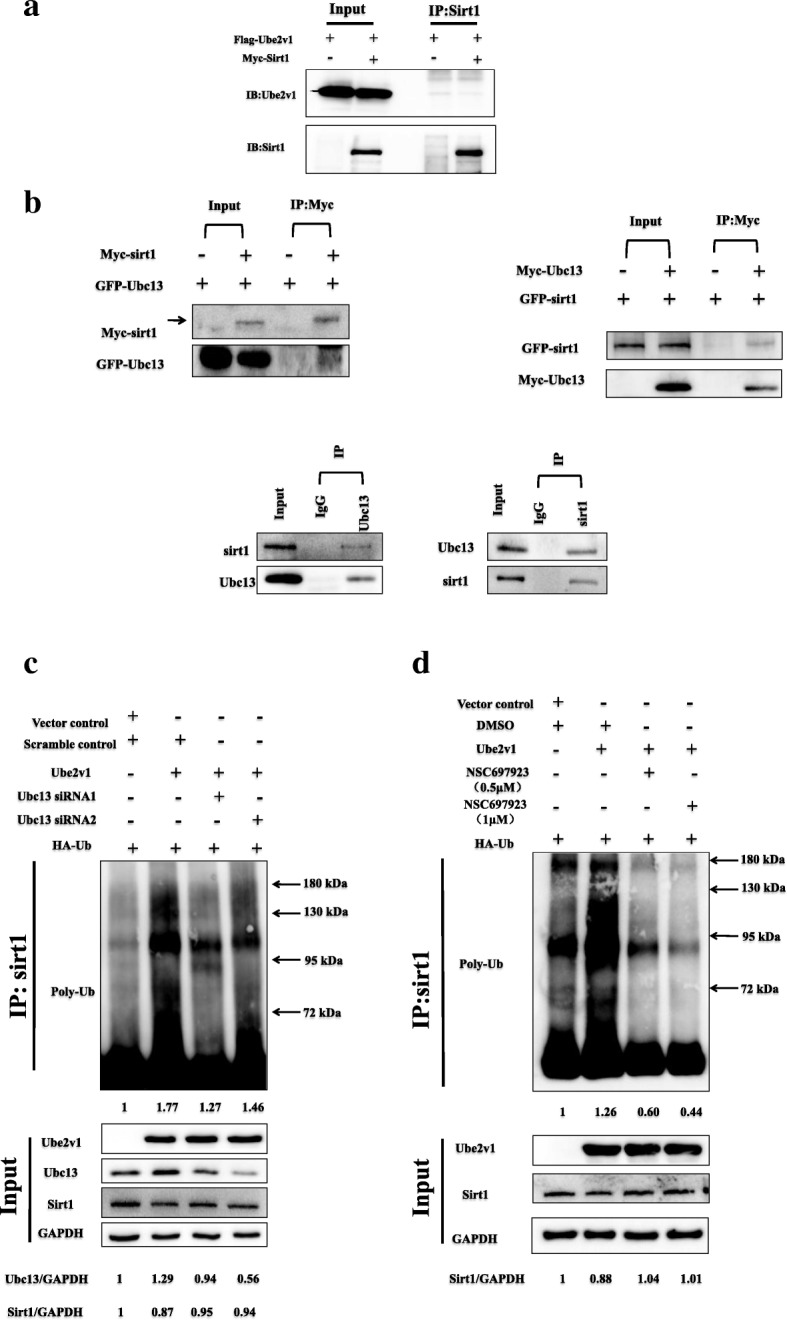
The role of Ubc13 in Ube2v1 mediates Sirt1 ubiquitination in CRC cells. **a** Immunoprecipitation assay for the exogenous interaction between Ube2v1 and Sirt1in SW480 cells transfected with Flag-Ube2v1 and Myc-Sirt1. **b** Immunoprecipitation assay for the exogenous and endogenous interaction between Ubc13 and Sirt1 in SW480 cells. **c** Ubiquitination assays of endogenousSirt1 in the lysates from SW480 cells cotransfected with GFP-Ube2v1, HA-Ub, two siRNAs targeting Ubc13, or vector control. The cells were treated with or without MG132 (20 μM) before harvest and then immunoprecipitated them with anti-Sirt1 antibody. **d** Ubiquitination assays of endogenous Sirt1 in the lysates from SW480 cells cotransfected with GFP-Ube2v1, HA-Ub, NSC697923 (a small-molecule inhibitor targeting Ubc13), or DMSO control. The cells were treated with or without MG132 (20 μM) before harvest and then immunoprecipitated them with anti-Sirt1 antibody

To study the underlying mechanisms by which Ube2v1 mediates Sirt1 ubiquitination, we examined whether there is an interaction between Ube2v1 and Sirt1. Co-immunoprecipitation assay showed that there was no direct interaction between Ube2v1 and Sirt1 in CRC cells (Fig. 3a). As Ube2v1 is a ubiquitin-conjugating E2 enzyme variant proteins without the conserved cysteine residue essential for the catalytic activity of E2s, the functions of Ube2v1 might be mediated by Ubc13 [33]. Interestingly, we found that Ubc13 can directly interact with Sirt1 in both exogenous and endogenous cell systems by co-immunoprecipitation (Fig. 3b). Functionally, knockdown of Ubc13 (Fig. 3c) or NSC697923 treatment (Fig. 3d), a small molecular inhibitor targeting interaction between Ubc13 and Ube2v1, can effectively attenuate the Sirt1 ubiquitination, demonstrating that Ubc13 is required for the regulatory effects of Ube2v1 on Sirt1 ubiquitination.

### Ube2v1 promotes epithelial mesenchymal transition by suppressing autophagy program in CRC cells

The role of autophagy in cancer is very complex, depending on context and types of cancers [34, 35]. Epithelial mesenchymal transition (EMT) has been implicated to play a key role in tumor progression and metastasis [36]. Whether Ube2v1 is involved in EMT through autophagy program is unknown.

**Fig. 4:**
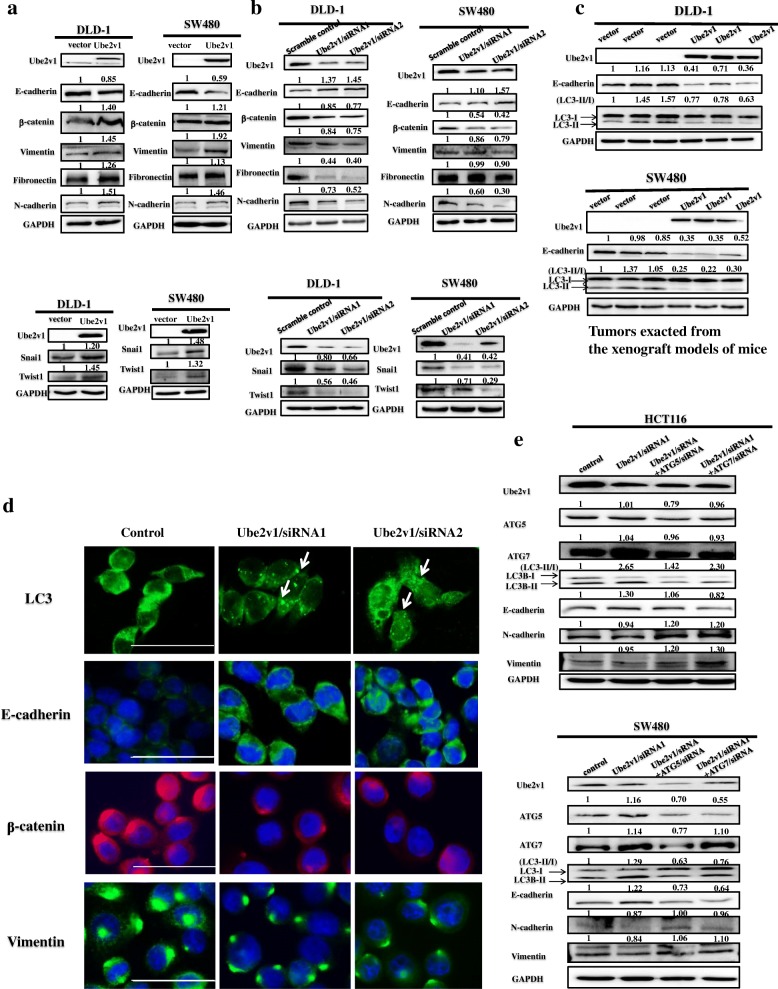
The effects of Ube2v1 on epithelial mesenchymal transition and autophagy program in colorectal cancer. **a**, **b** Expressions of E-cadherin, β-catenin, Vimentin, Fibronectin, N-cadherin, Twist1, and Snai1were detected after Ube2v1 was overexpressed (**a**) or knocked down (**b**) in DLD-1 and SW480 cells. **c** Tumor exacted from mouse xenograft model were used to detect expressions of E-cadherin and LC3-II in both DLD-1 and SW480 cells with Ube2v1 stable overexpression by western blotting. **d** Endogenous LC3 puncta and expressions of E-cadherin, β-catenin, and Vimentin were observed after Ube2v1 was knocked down in SW480cells by immunofluorescent analysis. **e** Expressions of Ube2v1, ATG5, ATG7,LC3-II, E-cadherin, β-catenin, Vimentin, Fibronectin, and N-cadherin were observed after Ube2v1 was knocked down in HCT116 and SW480 cells with or without knockdown of ATG5 or ATG7 using RNA interference

We found that Ube2v1 promotes EMT of CRC cells as demonstrated by decreased expression of E-cadherin, as well as increased expressions of β-catenin, Vimentin, Fibronectin, N-cadherin, Snai1, and Twist1 following Ube2v1 overexpression in DLD-1 and SW480 cells (Fig. 4a). Consistently, increased expression of E-cadherin and decreased expressions of β-catenin, Vimentin, Fibronectin, N-cadherin, Snai1, and Twist1 were found after Ube2v1was knocked down in DLD-1 and SW480 cells (Fig. 4b).

To explore the potential link between EMT and autophagy program, we used tumor samples from mouse xenograft model for western blotting and immunohistochemical staining. Western blots showed that decreased expression of E-cadherin and LC3-II was found in both DLD-1 and SW480 cells with Ube2v1 stable overexpression (Fig. 4c). In addition, immunofluorescence results indicated that increased endogenous LC3 puncta and E-cadherin was found after Ube2v1 was knocked down in SW480 cells (Fig. 4d), suggesting the link between EMT and autophagy program.

To further establish the regulatory effects of autophagy program mediated by Ube2v1 on EMT, inhibition of autophagy program by silencing expression of ATG5 or ATG7 can successfully rescued the suppressive effects on EMT initiated by Ube2v1 knockdown in HCT116 and SW480 cells (Fig. 4e). Additionally, 3-methyladenine (3-MA), an autophagy inhibitor, can effectively abolish the enhanced expression of E-cadherin induced by Ube2v1 knockdown (Additional file 3: Figure S4).

### Rapamycin attenuates the Ube2v1-mediated migration and invasion in vitro and lung metastasis of CRC cells in an orthotropic mouse xenograft model by restoring autophagy program

To further study the functional role of Ube2v1 in CRC, the expression levels of Ube2v1 in CRC cell lines were inspected at first and we found that SW480 and DLD-1 expressed low levels of Ube2v1. Therefore, Ube2v1 was stably overexpressed in SW480 and DLD-1 cells using a lentiviral system. The expression efficiency was confirmed at both mRNA and protein levels by Western blotting and qRT-PCR (data not showed). Scratch-wound healing assays showed that overexpression of Ube2v1 significantly enhanced CRC cell migration (Additional file 3: Figure S5A), consistent with the observations in the migration assay after Ube2v1 was overexpressed (Additional file 3: Figure S5B). Similarly, invasion assays indicated enhanced invasion ability for cells with Ube2v1 stable overexpression (Additional file 3: Figure S5C). Furthermore, we found that treatment with rapamycin, an agonist of autophagy, can successfully alleviate the increased migration and invasion abilities of CRC cells after Ube2v1 overexpression (Additional file 3: Figure S6).

**Fig. 5:**
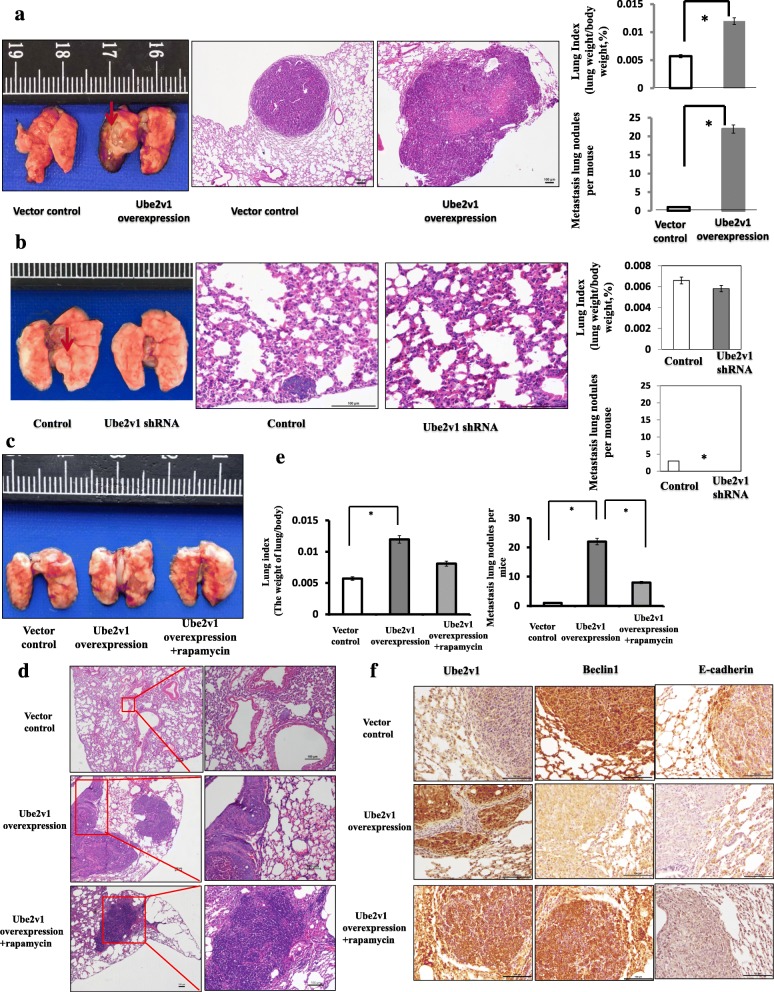
Effects of rapamycin on Ube2v1-mediated lung metastasis in the orthotropic mouse xenograft models of CRC. **a** Lung index and numbers of metastatic lung nodules of mice administrated intravenously with SW480 cells stably overexpressing Ube2v1 (2 × 10^6^ cells) were determined (*n* = 6 per group). **b** Lung index and numbers of metastatic lung nodules of mice administrated intravenously with SW480 cells stably knocked down expression of Ube2v1 (2 × 10^6^ cells) were determined (*n* = 6 per group). Representative photographs of the lung with metastatic nodules are shown (arrow heads). Representative micrographs of the lung with metastatic cells are shown by H&E staining at a magnification of × 200. M metastatic lesion, N adjacent normal lung tissue. Expressions of E-cadherin and LC3-II were detected after Ube2v1 was overexpressed with or without rapamycin (final concentration 10 ng/ml) stimulation. **c** Representative photographs of the lung with metastatic nodules are shown. **d** Representative micrographs of the lung with metastatic cells are shown by H&E staining at a magnification of × 100 (left) and × 200 (right). **e** Lung index and numbers of metastatic lung nodules of mice were determined (*n* = 6 per group). M metastatic lesion, N adjacent normal lung tissue. Error bars represent mean ± s.d. Statistical significance was determined by a two-tailed, unpaired Student’s *t* test. *p < 0.05, **p < 0.01. **f** Expressions of Ube2v1, Beclin1, and E-cadherin were analyzed by immunohistochemical staining. Error bars represent mean ± s.d. Statistical significance was determined by a two-tailed, unpaired Student’s *t* test. *p < 0.05

Lung metastasis models were used to monitor the effects of Ube2v1 overexpression or knockdown on metastasis of CRC in vivo. SW480 cells with Ube2v1 stable overexpression or knockdown were respectively injected into the lateral vein in the tails of the nude BALB/c mice. Five weeks after implantation, the lung metastatic index in mice administrated with SW480 cells with Ube2v1 stable overexpression increased significantly compared with those mice administrated with control SW480 cells (Fig. 5a). Histological examination showed SW480 cells with Ube2v1 stable overexpression were found to develop more and larger micrometastases in the lung of mice than control cells (Fig. 5a). In accordance with these results, knockdown of Ube2v1 resulted in less and smaller micrometastases in the lung (Fig. 5b). More importantly, rapamycin, an agonist of autophagy, can rescue the enhanced effects of lung metastasis induced by Ube2v1 overexpression in vivo (Fig. 5c–e). Immunohistochemical staining indicated that Beclin1 expression was significantly restored, accompanied by increased expressions of E-cadherin after administration of rapamycin (Fig. 5f).

Collectively, Ube2v1 promotes EMT and metastasis of CRC in an autophagy-dependent mechanism in vitro and in vivo.

### High Ube2v1 expression in CRC samples is correlated with poorer survival of CRC patients

**Fig. 6:**
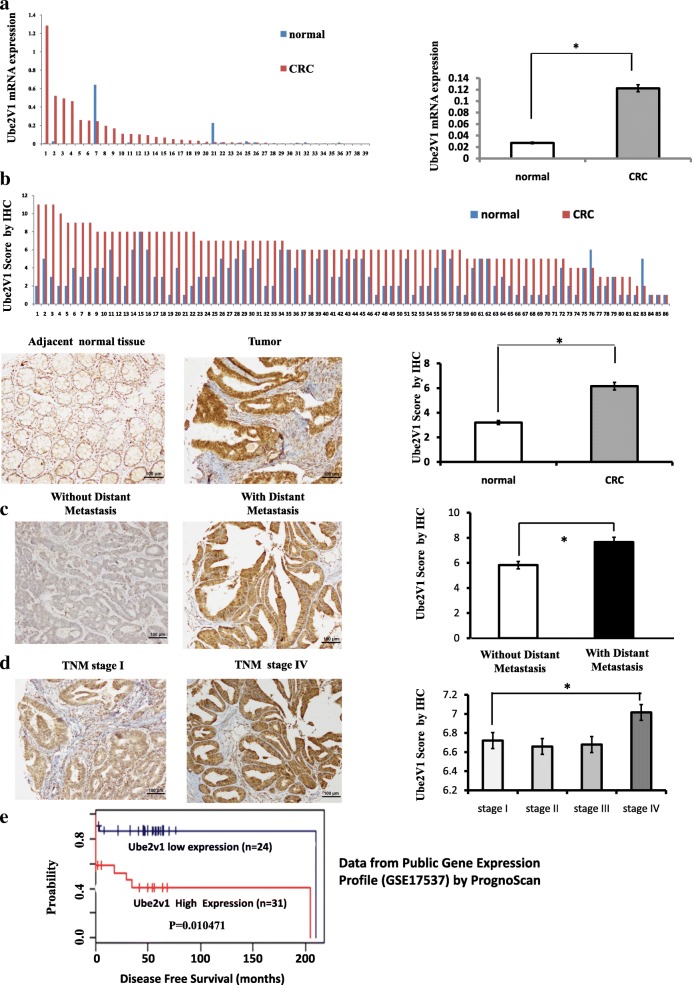
Ube2v1 expression in clinical samples of colorectal cancer. **a** mRNA levels of Ube2v1 in a tumor and its adjacent tissue from a CRC patient using qPCR. **b** Scores for Ube2v1 staining in paired, adjacent normal and tumor tissue samples from CRC patients by immunohistochemical staining. Typical staining for Ube2v1 in paired, adjacent normal and tumor tissue sample from a CRC patient. **c** Scores for Ube2v1 staining in tumor samples from CRC patients with or without distant metastasis by immunohistochemical staining. **d** Scores for Ube2v1 staining in tumor samples from CRC patients with different TNM stages by immunohistochemical staining. Typical staining for Ube2v1 in tumor samples from CRC patients in TNM stage I and stage IV was shown. **e** Disease-free survival of CRC patients according to the expression levels of Ube2v1 mRNA by PrognoScan using data from Public Gene Expression Profile (GSE17537). Statistical significance was determined by using a two-tailed, unpaired Student’s *t* test. *p < 0.05

Finally, the clinical significance of Ube2v1 in CRC was determined by analyzing the mRNA and protein levels of Ube2v1 in CRC patient samples. At both mRNA and protein levels, Ube2v1 expression was significantly increased in primary CRC tissue samples compared with that in their normal counterparts (Fig. 6a, b). More interestingly, Ube2v1 expression was markedly increased in CRC patients with distant metastases (Fig. 6c) or advanced clinical stage such as TNM stage IV when compared with that in patients without distant metastases or early clinical stage such as TNM stage I (Fig. 6d). Thus, the Ube2v1 exhibits a stage-specific expression which is correlated with CRC progression. Using data from Public Gene Expression Profile (GSE17537), meta-analysis of the prognostic value of Ube2v1 mRNA by PrognoScan showed that CRC patients with high levels of Ube2v1 mRNA might have shorter disease-free survival (Fig. 6e).

## Discussion

Ube2v1 (also named as Uev1A), one of ubiquitin-conjugating E2 enzyme variant proteins, belongs to a distinct subfamily of the E2 protein family [20, 37]. Based on its structure characteristics, Ube2v1 may have unknown functions different from those classic E2s.

Here, we present a critical role of Ube2v1 in autophagy program. We found Ube2v1 can epigenetically regulate autophagy genes. Ube2v1 can globally suppress gene expression of autophagy-related genes. Mechanistically, Ube2v1 promotes ubiquitination and degradation of Sirt1 through Ubc13, subsequently reduces H4K16ac, and suppresses gene expression of autophagy genes epigenetically. It has been shown that H4K16ac is one of the primary histone target of Sirt1 which is a key epigenetic player involved in autophagy. Our study provided a novel mechanistic understanding about the functional regulation of Sirt1 by Ube2v1-Ubc13-mediated ubiquitination. Noteworthily, our study link the classic ubiquitin-proteasome system especially the E2 member with autophagy machine in CRC, suggesting cooperative interaction between these two systems to control cellular homeostasis in CRC. For a long time, Ube2v1-UBC13, as a key components working with TRAF6, controls NF-κB signaling pathways. Recent reports showed that TAK1 kinase and OTUB1 controls NF-κB activation using similar Ube2v1-UBC13/TRAF6 signaling axis. The understanding of Ube2v1-UBC13 in autophagy discovered a new regulatory pathway for autophagy.

Moreover, we also uncovered a key mechanism by which Ube2v1 promotes metastasis of CRC cells. Accumulating evidences demonstrated that cancer cells usually undergo EMT program to facilitate invasion and metastasis [36, 38–40]. Ube2v1 promotes an autophagy-dependent EMT of CRC cells in vitro and in vivo. Our study functionally links the EMT with autophagy program in CRC. Nevertheless, the role of autophagy in cancer is very complex, depending on cell types and specific process involved [10, 18, 41–44]. In general, tumor cell autophagy has evolved to deal with intracellular and environmental stress, thus favoring tumor growth and progression [43]. This implies that autophagy may have differential impact in distinct phases of tumorigenesis including metastasis. For example, the role of autophagy in EMT processes is also diverse. Autophagy induction impairs migration and invasion by reversing EMT in glioblastoma cells [45]. On the contrary, Beclin1 overexpression promoted EMT process through Wnt/β-catenin pathway under starvation [46]. Moreover, the pan-inhibitor of Aurora kinases danusertib induces autophagy and suppresses EMT in human breast cancer cells [47]. Autophagy deficiency stabilizes Twist1 to promote EMT [48]. DEDD interacts with PI3KC3 to activate autophagy and attenuate EMT in human breast cancer [49, 50]. In liver-specific autophagy-deficient mice (Alb-Cre; ATG7(fl/fl)), autophagy deficiency in vivo reduces epithelial markers’ expression and increases the levels of mesenchymal markers [51]. In this study, we found that the tumor cells survived and metastasized to distant organs by a suppressive autophagy program.

Our study presented here has illuminated pro-metastatic function of Ube2v1 in CRC. We present a critical role of Ube2v1 in tumor growth and metastasis of CRC in vivo and in vitro. In CRC patients, Ube2v1 expression is elevated in tumor samples especially in advanced TNM staging and correlated with poorer survival of patients. In vivo studies using orthotopic mouse xenograft models of CRC showed that Ube2v1 promotes tumor growth and metastasis. To our knowledge, the role of Ube2v1 in CRC progression and metastasis has not been established. Only several papers have reported its controversial roles in cancer. Ube2v1, first named as CROC-1, was suggesting as a candidate oncogene by transcriptionally activating FOS proto-oncogene [19]. Inconsistently, Ube2v1 can also function as a tumor suppressor by protecting cells from DNA damage [21]. And Ube2v1 expression is increased significantly in the early stage for the acquisition of immortality of tumor cells [22]. Recently, Ube2v1 was reported to mediate matrix metalloproteinase-1 gene regulation through nuclear factor-кB and promote breast cancer metastasis [23]. Although concrete evidence for the role of Ube2v1 in cancer remains, some inhibitors targeting Ube2v1 pathway have been developed to treat some type of cancers, such as diffuse large B cell lymphoma cells [33]. Our study presented here has paved the path forward to develop small-molecule inhibitor targeting Ube2v1 for CRC treatment.

## Conclusion

Ube2v1 suppresses autophagy program and promotes metastasis of CRC by an autophagy-related EMT mechanism (Additional file 3: Figure S7), shedding light on developing Ube2v1 as a potential target for CRC treatment.

## Additional files

Additional file 1: Identification information of cell lines. (PDF 2605 kb)

Additional file 2: **Table S1.** Primers for qPCR or DNA sequencing. **Table S2.** Differential proteins between control cells and cells with Ube2v1 stable overexpression by quantitative mass spectrometry. (PDF 107 kb)

Additional file 3: **Figure S1.** Protein expressions of LC3-II and Beclin1 were examined by western blots when Ube2v1 was overexpressed in DLD-1 and SW480 cells under both normal medium culture condition (a) and starvation in Hank’s buffered saline solution (HBSS) (b). The cells were treated with Bafilomycin A1(BafA1), an autophagy inhibitor which blocks autophagosome–lysosome fusion. **Figure S2.** The effects of Ube2v1 on gene expressions of autophagy genes. A-B. mRNA levels of autophagy genes (LC3, Beclin1, ATG16L1, ATG3, ATG5, ATG7, ATG12, ATG10, ATG4a, ATG4b, ATG4c and ATG4d) were detected by qPCR analysis when Ube2v1 expression was knocked down (a) or overexpressed (b) in SW480 cells. C-D. mRNA levels of autophagy genes (LC3, Beclin1, ATG16L1, ATG3, ATG5, ATG7, ATG12, ATG10, ATG4a, ATG4b, ATG4c and ATG4d) were detected by qPCR analysis when Sirt1 expression was knocked down (c) or overexpressed (d) in SW480 cells. **Figure S3.** The effects of Ube2v1 on stabilization and ubiquitination of Sirt1 in CRC cells. The expression of Sirt1 was detected by western blotting in shRNA-transduced cells (a) or Ube2v1 overexpressed (b) SW480 cells treated with cyclohexamide (CHX) (100 μg/ml) for the indicated time intervals. The intensity of endogenous Sirt1 expression for each time point was quantified by densitometry. c Ubiquitination assays of exogenous Sirt1 in the lysates from SW480 cells cotransfected with GFP-Ube2v1, HA-Ub, Myc-Sirt1 or vector control. The cells were treated with or without MG132 (20 μM) before harvest and then immunoprecipitated them with anti-myc antibody. d Ubiquitination assays of exogenous Sirt1 in the lysates from SW480 cells cotransfected with HA-Ub, Myc-Sirt1 in the lysates from SW480 cells stably expressing Ube2v1 shRNA(shRNA/Ube2v1) or shRNA (shRNA/Control). **Figure S4.** Expressions of E-cadherin after Ube2v1 knockdown with stimulation of Autophagy inhibitor, 3-Methyladenine (3-MA) (5 mM) for 24 h. **Figure S5.** The effects of Ube2v1overexpression on wound-healing, migration and invasion of CRC cells. Wound healing (a), migration (b) and invasion (c) of SW480 and DLD-1 cells after Ube2v1 expression was stably overexpressed. Statistical significance was determined by using a two-tailed, unpaired student’s t-test.*p < 0.05. **Figure S6.**The effects of rapamycin on Ube2v1 mediated in vitro migration and invasion of CRC cells. a Expressions of E-cadherin and LC3-II, were detected after Ube2v1 was overexpressed with or without rapamycin (final concentration:10 ng/ml) stimulation. B-C. In vitro migration (b) and invasion (c) assays of CRC cells after Ube2v1 overexpression with or without rapamycin (final concentration:10 ng/ml) stimulation for 36 h. **Figure S7.** A proposed model for the role of Ube2v1 in CRC metastasis. Ube2v1 promotes ubiquitination and degradation of Sirt1 by the help of Ubc13, reduces H4K16ac, and finally epigenetically suppresses gene expression of autophagy genes. Then, Ube2v1 promotes autophagy-dependent EMT and metastasis in CRC. (PDF 2486 kb)

## Abbreviations

3-MA: 3-Methyladenine
CHX: Cycloheximide
CRC: Colorectal cancer
EMT: Epithelial mesenchymal transition
H4K16ac: Histone H4 lysine 16 acetylation
Sirt1: Sirtuin 1
Ube2v1: Ubiquitin-conjugating E2 enzyme variant 1
