# Supplementary material for: Ube2v1-mediated ubiquitination and degradation of Sirt1 promotes metastasis of colorectal cancer by epigenetically suppressing autophagy
Source: J Hematol Oncol. 2018 Jul 17;11:95. doi: 10.1186/s13045-018-0638-9 (PMC6050692; doi:10.1186/s13045-018-0638-9)
Supplement: Supplementary file 1 — Identification information of cell lines. (PDF 2605 kb) [file 13045_2018_638_MOESM1_ESM.pdf]

Report of Human Cell Line Authentication

（Notice:This authentication report is restricted to the cell sold from Guangzhou Cellcook Biotech Co., Ltd, and the date with seal is the date of delivery. ）

Ⅰ. Sample

Sample Name: labeled as ‘SW480’

Ⅱ. Method and Procedure

1. PCR is amplified with STR Multi-amplification Kit (PowerPlex^{TM}16HS System);
2. PCR products are assayed with 3100 DNA Analyzer (Applied Biosystems®).
3. Amplification of gene COX1 and electrophoresis are employed to survey the species of the sample.

Ⅲ. Results

1. The STR profiles of the cell line sample are in the attached table and figure.
2. The search result in ATCC and DSMZ databases.
3. The electrophoresis figure of gene COX1.

SW480: ①No loci has tri-alleles or tetra-alleles. Contamination of other human cell line is not found (Figure 1 & Table 1). ②100% matched cell lines are found in ATCC and DSMZ data banks. And the cell line named as “SW480” *et al*. (Figure 2 & Figure 3) ③The sample is a human cell line. Contamination of other species cells are not found in the sample (Figure 4).

Operator: Xiaohua Mo

Auditor: Xuanyi Liang

Guangzhou Cellcook Biotech Co., Ltd

GUANGZHOU CELLCOOK BIOTECH CO.,LTD
[illegible] JAN 2018
CELL STR PROFILING
PASS

Figure 1. STR profiles of SW480 cell line

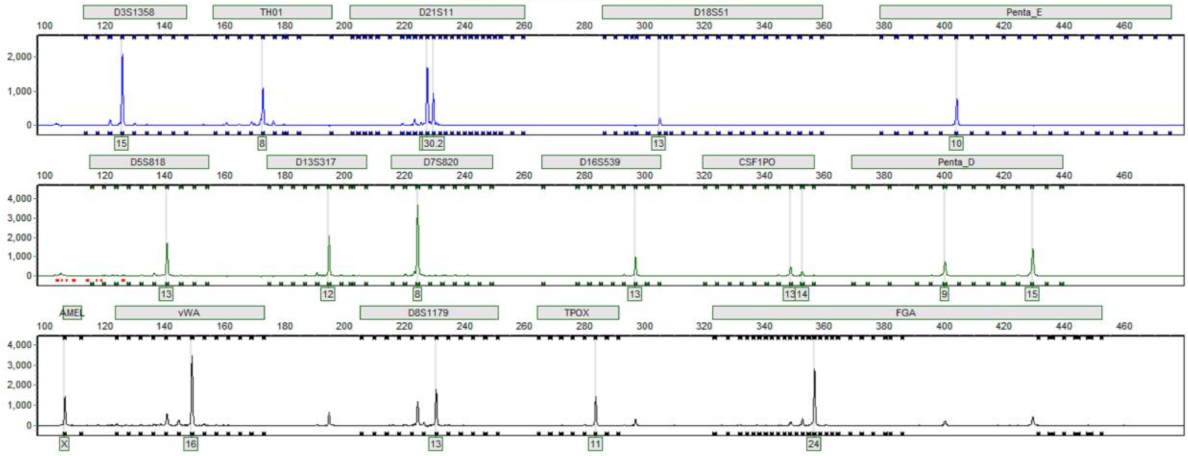

Table 1. STR profiles of SW480 cell line

	Allele1	Allele2
D3S1358	15	
TH01	8	
D21S11	30	30.2
D18S51	13	
Penta_E	10	
D5S818	13	
D13S317	12	
D7S820	8	
D16S539	13	
CSF1PO	13	14
Penta_D	9	15
AMEL	x	
vWA	16	
D8S1179	13	
TPOX	11	
FGA	24	

Figure 2. Search result in ATCC database

SEARCH THE STR DATABASE

As part of our continuing efforts to characterize and authenticate the cell lines in the Cell Biology collection, ATCC has developed a comprehensive database of short tandem repeat (STR) DNA profiles for all of our human cell lines. View our brief tutorial before starting.

1. STR Profiling Analysis
2. Matching Algorithm
3. Interrogating the Database

Showing 1 - 3 Of 3 PageSize: 100

Add to Cart	%Match	ATCC® Number	Designation	D5S818	D13S317	D7S820	D16S539	vWA	TH01	AMEL	TPOX	CSF1PO
☐	83.33	CCL-227	SW-620	13	12	8,9	9,13	16	8	X	11	13,14
☐	100	CCL-228	SW-480	13	12	8	13	16	8	X	11	13,14
☐	90.91	CRL-7940	SW 527	13	12	8	9,13	16	8	X	11	13,14

Add to Cart Export to Excel

Figure 3. Search result in DSMZ database

Result of STR matching analysis by your data.

- DSMZ Profile Database -

A graphical presentation is shown at the bottom of this page.

EV	Cell No.	Cell name	Locus names									Figures
			D5S818	D13S317	D7S820	D16S539	VWA	TH01	AM	TPOX	CSF1PO	
	Query (Your Cell)		*13*	*12*	*8*	*13*	*16*	*8*	*x*	*11*	*13,14*	
1.29(36/28)	313	SW-480	13,13	12,12	8,8	13,13	16,16	8,8	X,X	11,11	13,14	-
1.29(36/28)	CCL-228	SW480 [SW-480]	13,13	12,12	8,8	13,13	16,16	8,8	X,X	11,11	13,14	-
1.29(36/28)	CRL-2176	SW-598	13,13	12,12	8,8	13,13	16,16	8,8	X,X	11,11	13,14	-
1.21(34/28)	CRL-7940	SW 527	13,13	12,12	8,8	9,13	16,16	8,8	X,X	11,11	13,14	-
1.14(32/28)	CCL-227	SW620 [SW-620]	13,13	12,12	8,9	9,13	16,16	8,8	X,X	11,11	13,14	-
0.86(24/28)	JCRB1031	JHH-7	13,13	12,12	8,11	13,13	16,18	6,6	X,Y	9,11	13,14	-
0.79(22/28)	CRL-2547	Panc 10.05	13,13	12,12	8,9	9,12	16,16	6,9.3	X,X	11,11	12,12	-
0.79(22/28)	CRL-2558	PL45	13,13	12,12	8,9	9,12	16,16	6,9.3	X,X	11,11	12,12	-
0.71(20/28)	680	NALM-16	11,11	12,12	10,10	13,13	17,17	8,8	X,X	11,11	11,11	-
0.71(20/28)	CRL-1848	NCI-H292 [H292]	13,13	11,12	10,10	9,13	16,17	8,8	X,X	8,11	10,10	-
[illegible]	[illegible]	[illegible]	[illegible]	[illegible]	[illegible]	[illegible]	[illegible]	[illegible]	[illegible]	[illegible]	[illegible]	[illegible]

Figure 4. Authentication of the species of the sample

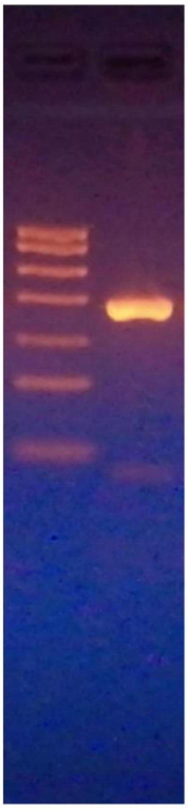

M: Marker. As the size of 700, 600, 500, 400, 300, 200 and 100bp from up to down.

Nine species are checked, as follow: *Homo sapiens* 391bp, *Cricetulus griseus*315bp, *Macaca mulatta*287bp, *Cercopithecus aethiops*222bp, *Rattus norvegicus*196bp, *Canis familiaris*172bp, *Mus musculus*150bp, *Bos Taurus*102bp, IC 70bp

The sample: The band size is 391bp which matches the size of human.

Report of Human Cell Line Authentication

（Notice:This authentication report is restricted to the cell sold from Guangzhou Cellcook Biotech Co., Ltd, and the date with seal is the date of delivery. ）

Ⅰ. Sample

Sample Name: labeled as ‘HCT-116’

Ⅱ. Method and Procedure

1. PCR is amplified with STR Multi-amplification Kit (PowerPlex^{TM}16HS System);
2. PCR products are assayed with 3100 DNA Analyzer (Applied Biosystems®).
3. Amplification of gene COX1 and electrophoresis are employed to survey the species of the sample.

Ⅲ. Results

1. The STR profiles of the cell line sample are in the attached table and figure.
2. The search result in ATCC and DSMZ databases.
3. The electrophoresis figure of gene COX1.

HCT-116: ①One loci has tri-alleles (D3S1358). Contamination of other human cell line is not found (Figure 1 & Table 1). ②100% matched cell lines are found in ATCC and DSMZ data banks. And the cell line named as “HCT-116” *et al*. (Figure 2 & Figure 3) ③The sample is a human cell line. Contamination of other species cells are not found in the sample (Figure 4).

Operator: Xiaohua Mo

Auditor: Xuanyi Liang

Guangzhou Cellcook Biotech Co., Ltd

GUANGZHOU CELLCOOK BIOTECH CO.,LTD
12 DEC 2017
CELL STR PROFILING
PASS

Figure 1. STR profiles of HCT-116 cell line

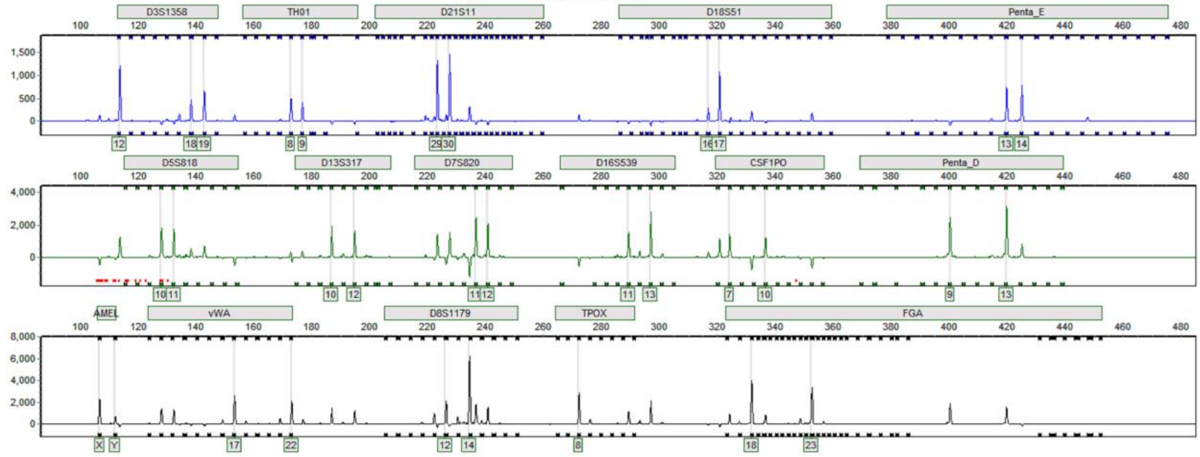

Table 1. STR profiles of HCT-116 cell line

	Allele1	Allele2	Allele3
D3S1358	12	18	19
TH01	8	9	
D21S11	29	30	
D18S51	16	17	
Penta_E	13	14	
D5S818	10	11	
D13S317	10	12	
D7S820	11	12	
D16S539	11	13	
CSF1PO	7	10	
Penta_D	9	13	
AMEL	x	Y	
vWA	17	22	
D8S1179	12	14	
TPOX	8		
FGA	18	23	

Figure 2. Search result in ATCC database

SEARCH THE STR DATABASE

As part of our continuing efforts to characterize and authenticate the cell lines in the Cell Biology collection, ATCC has developed a comprehensive database of short tandem repeat (STR) DNA profiles for all of our human cell lines. View our brief tutorial before starting.

1. STR Profiling Analysis
2. Matching Algorithm
3. Interrogating the Database

Showing 1 - 2 Of 2 PageSize: 100

Add to Cart	%Match	ATCC® Number	Designation	D5S818	D13S317	D7S820	D16S539	vWA	TH01	AMEL	TPOX	CSF1PO
☐	100	CCL-247	HCT 116	10,11	10,12	11,12	11,13	17,22	8,9	X,Y	8,9	7,10
☐	87.5	CRL-2780	ATRFLOX	10,12	10,12	11,12	11,13	17,22	8,9	X	8	7,9

Add to Cart Export to Excel

Disclaimer: Reference to this database and the data contained therein may be cited in publications, and ATCC encourages such citation or reference. While every reasonable effort has been made to assure the accuracy of these data, no warranty, express or implied, is made by ATCC as to their accuracy.

Figure 3. Search result in DSMZ database

Result of STR matching analysis by your data.

- DSMZ Profile Database -

A graphical presentation is shown at the bottom of this page.

EV	Cell No.	Cell name	Locus names									Figures
			D5S818	D13S317	D7S820	D16S539	VWA	TH01	AM	TPOX	CSF1PO	
	Query (Your Cell)		*10,11*	*10,12*	*11,12*	*11,13*	*17,22*	*8,9*	*X,Y*	*8,9*	*7,10*	
1.00(36/36)	CCL-247	HCT 116	10,11	10,12	11,12	11,13	17,22	8,9	X,Y	8,9	7,10	-
0.95(36/38)	581	HCT-116	10,11	10,12	11,12	11,13,12	17,17,21	8,8	X,X	8,8	7,10	-
0.89(32/36)	CRL-2780	ATRFLOX [Mutatect]	10,12	10,12	11,12	11,13	17,22	8,9	X,X	8,8	7,9	-
0.83(30/36)	CRL-1918	CFPAC-1	10,11	12,12	8,10	9,11	17,17	8,8	X,Y	8,8	10,10	-
0.78(28/36)	CRL-1997	HPAF-II	11,13	12,12	10,13	11,13	17,17	9,9	X,X	8,8	10,11	-
0.78(28/36)	CRL-5915	NCI-H2052 [H2052]	11,11	12,12	12,12	11,13	17,17	7,9.3	X,X	8,11	10,11	-
0.78(28/36)	RCB2266	HE50	11,12	10,12	8,9	11,11	17,17	9,9	X,Y	8,9	10,11	-
0.78(28/36)	CRL-5963	NCI-BL2052	11,11	12,12	11,12	11,13	17,17	7,9.3	X,Y	8,11	10,11	-
0.76(28/37)	CRL-5874	NCI-H1522 [H1522]	11,11	12,12	9,11,12	11,11	17,20	7,9.3	X,Y	8,11	10,10	-
0.76(28/37)	CRL-5873	NCI-H1514	11,11	12,12	9,12	11,11	17,21,22	7,9.3	X,Y	8,11	10,10	-
0.72(26/36)	104	M-07e	11,11	10,11	11,11	11,11	16,18	6,8	X,X	8,8	9,10	-

Figure 4. Authentication of the species of the sample

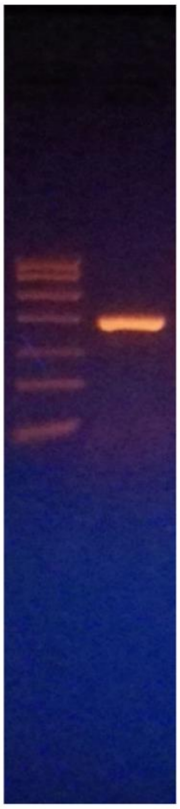

M: Marker. As the size of 700, 600, 500, 400, 300, 200 and 100bp from up to down.

Nine species are checked, as follow: *Homo sapiens* 391bp, *Cricetulus griseus*315bp, *Macaca mulatta*287bp, *Cercopithecus aethiops*222bp, *Rattus norvegicus*196bp, *Canis familiaris*172bp, *Mus musculus*150bp, *Bos Taurus*102bp, IC 70bp

The sample: The band size is 391bp which matches the size of human.

Report of Human Cell Line Authentication

（Notice:This authentication report is restricted to the cell sold from Guangzhou Cellcook Biotech Co., Ltd, and the date with seal is the date of delivery. ）

Ⅰ. Sample

Sample Name: labeled as 'DLD1'

Ⅱ. Method and Procedure

1. PCR is amplified with STR Multi-amplification Kit (PowerPlex^{TM}16HS System);
2. PCR products are assayed with 3100 DNA Analyzer (Applied Biosystems®).
3. Amplification of gene COX1 and electrophoresis are employed to survey the species of the sample.

Ⅲ. Results

1. The STR profiles of the cell line sample are in the attached table and figure.
2. The search result in ATCC and DSMZ databases.
3. The electrophoresis figure of gene COX1.

DLD1: ①No loci has tri-alleles or tetra-alleles. Contamination of other human cell line is not found (Figure 1 & Table 1). ②100% matched cell lines are found in ATCC and DSMZ data banks. And the cell line named as "DLD1" *et al*. (Figure 2 & Figure 3) ③The sample is a human cell line. Contamination of other species cells are not found in the sample (Figure 4).

Operator: Xiaohua Mo

Auditor: Xuanyi Liang

Guangzhou Cellcook Biotech Co., Ltd

GUANGZHOU CELLCOOK BIOTECH CO.,LTD
15 DEC 2017
CELL STR PROFILING PASS

Figure 1. STR profiles of DLD1 cell line

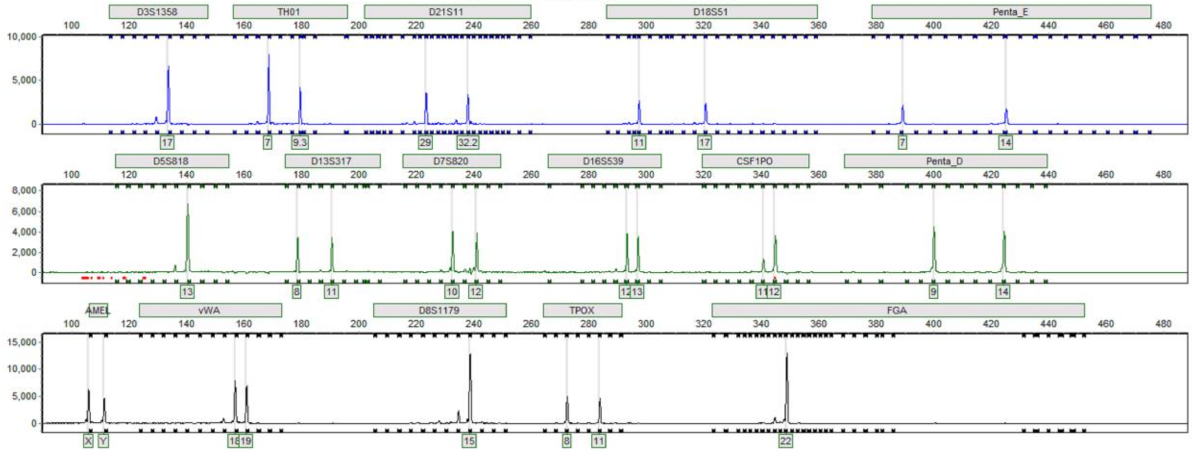

Table 1. STR profiles of DLD1 cell line

	Allele1	Allele2
D3S1358	17	
TH01	7	9.3
D21S11	29	32.2
D18S51	11	17
Penta_E	7	14
D5S818	13	
D13S317	8	11
D7S820	10	12
D16S539	12	13
CSF1PO	11	12
Penta_D	9	14
AMEL	x	y
vWA	18	19
D8S1179	15	
TPOX	8	11
FGA	22	

Figure 2. Search result in ATCC database

SEARCH THE STR DATABASE

As part of our continuing efforts to characterize and authenticate the cell lines in the Cell Biology collection, ATCC has developed a comprehensive database of short tandem repeat (STR) DNA profiles for all of our human cell lines. View our brief tutorial before starting.

1. STR Profiling Analysis
2. Matching Algorithm
3. Interrogating the Database

Showing 1 - 7 Of 7 PageSize: 100

Add to Cart	%Match	ATCC® Number	Designation	D5S818	D13S317	D7S820	D16S539	vWA	TH01	AMEL	TPOX	CSF1PO
☐	100	CCL-221	DLD-1	13	8,11	10,12	12,13	18,19	7,9.3	X,Y	8,11	11,12
☐	100	CCL-225	HCT-15	13	8,11	10,12	12,13	18,19	7,9.3	X,Y	8,11	12
☐	94.12	CCL-244	HCT-8	13	8,11	10,11.3,12	12,13	18,19	7,9.3	X,Y	8,11	12
☐	80	CRL-5838	NCI-H720	11	11	10,12	13	18	6	X	8	11
☐	81.82	CRL-5934	NCI-H2227	13	8	10,12	9	16,18	9.3	X	8	11
☐	83.33	CRL-5850	NCI-H920	13	11	10,11	11,13	19	9.3	X	8,11	12
☐	100	CRL-11663	HRT-18G	13	8,11	10,12	12,13	18,19	7,9.3	X,Y	8,11	12

Figure 3. Search result in DSMZ database

Result of STR matching analysis by your data.

- DSMZ Profile Database -

A graphical presentation is shown at the bottom of this page.

EV	Cell No.	Cell name	Locus names									Figures
			D5S818	D13S317	D7S820	D16S539	VWA	TH01	AM	TPOX	CSF1PO	
	Query (Your Cell)		*13*	*8,11*	*10,12*	*12,13*	*18,19*	*7,9.3*	*x,y*	*8,11*	*11,12*	
1.03(36/35)	278	DLD-1	13,13	8,11	10,12	12,13	18,19	7,9.3	X,Y	8,11	11,12	-
1.03(36/35)	357	HCT-15	13,13	8,11	10,12	12,13	18,19	7,9.3	X,Y	8,11	12,12	-
1.03(36/35)	CCL-221	DLD-1	13,13	8,11	10,12	12,13	18,19	7,9.3	X,Y	8,11	11,12	-
1.03(36/35)	CCL-225	HCT-15	13,13	8,11	10,12	12,13	18,19	7,9.3	X,Y	8,11	12,12	-
1.03(36/35)	JCRB9094	DLD-1	13,13	8,11	10,12	12,13	18,19	7,9.3	X,Y	8,11	11,12	-
1.03(36/35)	CRL-11663	HRT-18G	13,13	8,11	10,12	12,13	18,19	7,9.3	X,Y	8,11	12,12	-
1.00(36/36)	CCL-244	HCT-8 [HRT-18]	13,13	8,11	10,12,11.3	12,13	18,19	7,9.3	X,Y	8,11	12,12	-
0.91(32/35)	CRL-5850	NCI-H920 [H920]	13,13	11,11	10,11	11,13	19,19	9.3,9.3	X,X	8,11	12,12	-
0.86(30/35)	CRL-5934	NCI-H2227	13,13	8,8	10,12	9,9	16,18	9.3,9.3	X,X	8,8	11,11	-
0.80(28/35)	325	SNB-19	11,12	10,11	10,12	12,12	16,18	9.3,9.3	X,Y	8,8	11,12	-
[illegible]	[illegible]	[illegible]	[illegible]	[illegible]	[illegible]	[illegible]	[illegible]	[illegible]	[illegible]	[illegible]	[illegible]	[illegible]

Figure 4. Authentication of the species of the sample

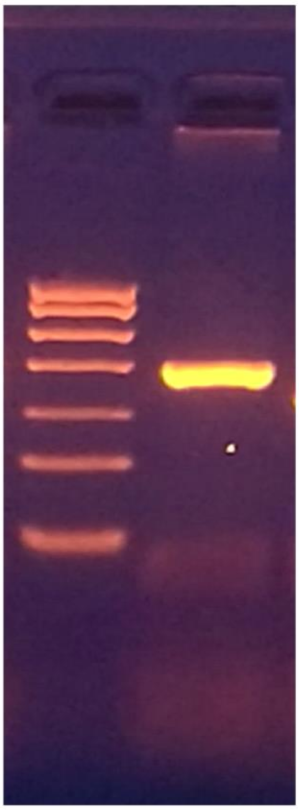

M: Marker. As the size of 700, 600, 500, 400, 300, 200 and 100bp from up to down.

Nine species are checked, as follow: *Homo sapiens* 391bp, *Cricetulus griseus*315bp, *Macaca mulatta*287bp, *Cercopithecus aethiops*222bp, *Rattus norvegicus*196bp, *Canis familiaris*172bp, *Mus musculus*150bp, *Bos Taurus*102bp, IC 70bp

The sample: The band size is 391bp which matches the size of human.
