# Supplementary material for: Ube2v1-mediated ubiquitination and degradation of Sirt1 promotes metastasis of colorectal cancer by epigenetically suppressing autophagy
Source: J Hematol Oncol. 2018 Jul 17;11:95. doi: 10.1186/s13045-018-0638-9 (PMC6050692; doi:10.1186/s13045-018-0638-9)
Supplement: Supplementary file 2 — Table S1. Primers for qPCR or DNA sequencing. Table S2. Differential proteins between control cells and cells with Ube2v1 stable overexpression by quantitative mass spectrometry. (PDF 107 kb) [file 13045_2018_638_MOESM2_ESM.pdf]

Table S1. Primers for qPCR or DNA sequencing

Genes	Primers
Ube2v1	F: AAAAGTCCCTCGCAATTTCC<br>R: CTGCCATTTTGCTAGCACTG
LC3	F: AGCAGCATCCAACCAAAATC<br>R: CTGTGTCCGTTCACCAACAG
Beclin1	F: GCCCAGACAGGACTCTCTTAG<br>R: TGAACACACTTGCCAGTCTTC
ATG16L1	F: ATGCGCGGATTGTCTCAGG<br>R: GTCCACTCATTACACATTGCTCT
ATG3	F: GATGGCGGATGGGTAGATACA<br>R: TCTTCACATAGTGCTGAGCAATC
ATG5	F: AAAGATGTGCTTCGAGATGTGT<br>R: CACTTTGTCAGTTACCAACGTCA
ATG7	F: ATGATCCCTGTAACTTAGCCCA<br>R: CACGGAAGCAAACAACTTCAAC
ATG12	F: TAGAGCGAACACGAACCATCC<br>R: CACTGCCAAAACACTCATAGAGA
ATG10	F: ATAAGATGCGACTGCTACAGGG<br>R: CAATGCTCAGCCATGATGTGAT
ATG4a	F: TGCTGGTTGGGGATGTATGC<br>R: GCGTTGGTATTCTTTGGGTTGT
ATG4b	F: GGTGTGGACAGATGATCTTTGC<br>R: CCAACTCCCATTTGCGCTATC
ATG4c	F: TAGAGGATCACGTAATTGCAGGA<br>R: GTTGTCAAAGCTGAGCCTTCTAT
ATG4d	F: CCAGCCCACTGTGGATGTC<br>R: AAGCCCACGGTACAGCTTG
Sirt1	F: CAGTGTCATGGTTCCTTTGC<br>R: CACCGAGGAACTACCTGAT
E-cadherin	F: TGAGTGTCCCCCGGTATCTTC<br>R: CAGTATCAGCCGCTTTCAGATTTT
N-cadherin	F: CACTGCTCAGGACCCAGAT<br>R: TAAGCCGAGTGATGGTCC
Vimentin	F: GAGAACTTTGCCGTTGAAGC<br>R: TCCAGCAGCTTCCTGTAGGT
Fibronectin	F:CCGTGGGCAACTCTGTC<br>R: TGCGGCAGTTGTCACAG
18sRNA	F: GGACCAGAGCGAAAGCATTTGCC<br>R: TCAATCTCGGGTGGCTGAACGC

Table S2. Differential proteins between control cells and cells with Ube2v1 stable overexpression by quantitative mass spectrometry.

Accession	Name	Peptides(95%)	Fold ratio	
			Control/Ube2v1	Ube2v1/Control
AAB08709	Prothymosin alpha	2	0.159	3.247
BAH00241	Ubiquitin-conjugating enzyme E2 variant 2	2	0.361	2.414
NP_002893	Reticulocalbin-2	1	0.554	1.737
NP_001093163	60S ribosomal protein L31	2	0.715	1.602
NP_001289978	Myosin regulatory light chain 12A	5	0.729	1.526
NP_001004	40S ribosomal protein S9	7	0.531	1.504

NP_001006	40S ribosomal protein S11	3	0.716	1.504
NP_001000	40S ribosomal protein S5	2	0.749	1.470
AAP97139	NSFL1 cofactor p47	1	0.757	1.466
EEB45048	cytochrome C	4	0.641	1.459
NP_036555	60S ribosomal protein L13a	4	0.799	1.409
NP_001949	Elongation factor 1-alpha 2	8	0.750	1.394
NP_000963	60S ribosomal protein L7a	8	0.780	1.368
NP_000981	60S ribosomal protein L27a	3	0.699	1.348
NP_001265518	Hsc70-interacting protein	5	0.765	1.345
AAD20951	Reticulon	1	0.706	1.336
NP_001020	40S ribosomal protein S26	1	0.745	1.317
NP_001003	40S ribosomal protein S8	5	0.747	1.284
NP_001020092	60S ribosomal protein L9	6	0.721	1.273

XP_544163	PREDICTED: copine-3	3	0.561	1.260
NP_001230059	Isoform 2 of 60S ribosomal protein L13	2	0.713	1.245
NP_001269316	isocitrate dehydrogenase [NADP] cytoplasmic	3	0.638	1.236
NP_001290554	60S ribosomal protein L10	6	0.795	1.228
NP_001001	40S ribosomal protein S6	5	0.800	1.218
XP_003478800	60S ribosomal protein L22-like	1	0.668	1.207
**Accession**	**Name**	**Peptides(95%)**	**Fold ratio**	
			**Control/Ube2v1**	**Ube2v1/Control**
NP_057215	Ras-related protein Rab-10	4	6.636	0.534

NP_003899	Eukaryotic translation initiation factor 2 subunit 2	1	5.175	0.000
NP_077305	EF-hand domain-containing protein D2	3	4.029	0.746
NP_000412	Keratin, type I cytoskeletal 10	2	3.564	0.769
AAA92155	Sorcin	1	3.084	0.000
NP_066402	Histone H2B type 1-J	3	2.943	0.481
AAN59974.1	Histone H2A	2	2.888	0.483
NP_778224.1	Histone H4	5	2.690	0.296
NP_003522.1	Histone H3.1	2	2.573	0.385
NP_001419	Isoform MBP-1 of Alpha-enolase	13	2.211	0.449
NP_006112	Keratin, type II cytoskeletal 1	2	2.178	0.563

NP_002087	General transcription factor IIF subunit 1	1	2.110	0.479
NP_003008.1	Serine/arginine-rich-splicing factor 3	2	2.068	0.619
NP_958844	Isoform 3 of Histone H2A.V	2	1.967	0.694
NP_056121	Ubiquitin carboxyl-terminal hydrolase 24	1	1.877	0.000
P23231	Mitochondrial import receptor subunit TOM70	1	1.835	0.000
NP_003065	SWI/SNF complex subunit SMARCC1	2	1.816	0.000
NP_001159579	Isoform 3 of Hydroxymethylglutaryl-CoA	1	1.707	0.000

	synthase, mitochondrial			
AAH35103	SNRPG protein	1	1.613	0.505
NP_060286	Cell growth-regulating nucleolar protein	1	1.605	0.000
NP_001164453	Isoform 3 of Heterogeneous nuclear ribonucleoproteins C1/C2	2	1.596	0.597
P02584	Profilin-1	3	1.596	0.000
NP_001244120	Probable ATP-dependent RNA helicase DDX6	1	1.565	0.682
NP_004044	Bystin	1	1.560	0.000
AAC39540	Heterogeneous nuclear ribonucleoprotein R	6	1.558	0.734

NP_004634	Polyadenylate-binding protein 2	1	1.543	0.000
NP_001257889	Nitric oxide synthase-interacting protein	1	1.540	0.000
NP_998776.1	Phosphate carrier protein, mitochondrial	1	1.537	0.000
AAH36708	Heterogeneous nuclear ribonucleoprotein A/B	4	1.520	0.577
NP_006342	Mitochondrial import inner membrane translocase subunit TIM44	3	1.506	0.674
NP_00117163	UDP-glucose 6-dehydrogenase isoform 3	1	1.495	0.656

NP_002128	heterogeneous nuclear ribonucleoproteins A2/B1 isoform A2	6	1.415	0.299
NP_542165	Spliceosome RNA helicase DDX39B	3	1.403	0.636
NP_001106674.2	Nascent polypeptide-associated complex subunit alpha, muscle-specific form	4	1.390	0.768
NP_055317	Pre-mRNA-processing factor 19	2	1.386	0.754
EAX02283	Aldehyde dehydrogenase 1 family, member A3, isoform	2	1.385	0.770

	CRA_b			
NP_001104547	Ezrin	21	1.379	0.702
P02769	Serum albumin	11	1.366	0.736
Q14978	Nucleolar and coiled-body phosphoprotein 1	3	1.363	0.539
NP_055969	LDLR chaperone MESD	3	1.354	0.716
NP_005110	Thyroid hormone receptor-associated protein 3	2	1.354	0.779
NP_001153147	heterogeneous nuclear ribonucleoprotein Q isoform 4	10	1.311	0.793
NP_005907	DNA replication licensing factor MCM7	4	1.289	0.716
NP_000692	sodium/potassium-transporting	2	1.285	0.646

	ATPase subunit alpha-1 isoform a			
NP_687033	proteasome subunit alpha type-3 isoform 2	2	1.282	0.756
P12763	Alpha-2-HS-glycoprotein precursor	3	1.241	0.787
NP_005773	THO complex subunit 4	3	1.220	0.699
