# Supplementary figures and images for: Ube2v1-mediated ubiquitination and degradation of Sirt1 promotes metastasis of colorectal cancer by epigenetically suppressing autophagy
Source: J Hematol Oncol. 2018 Jul 17;11:95. doi: 10.1186/s13045-018-0638-9 (PMC6050692; doi:10.1186/s13045-018-0638-9)

Figure S1

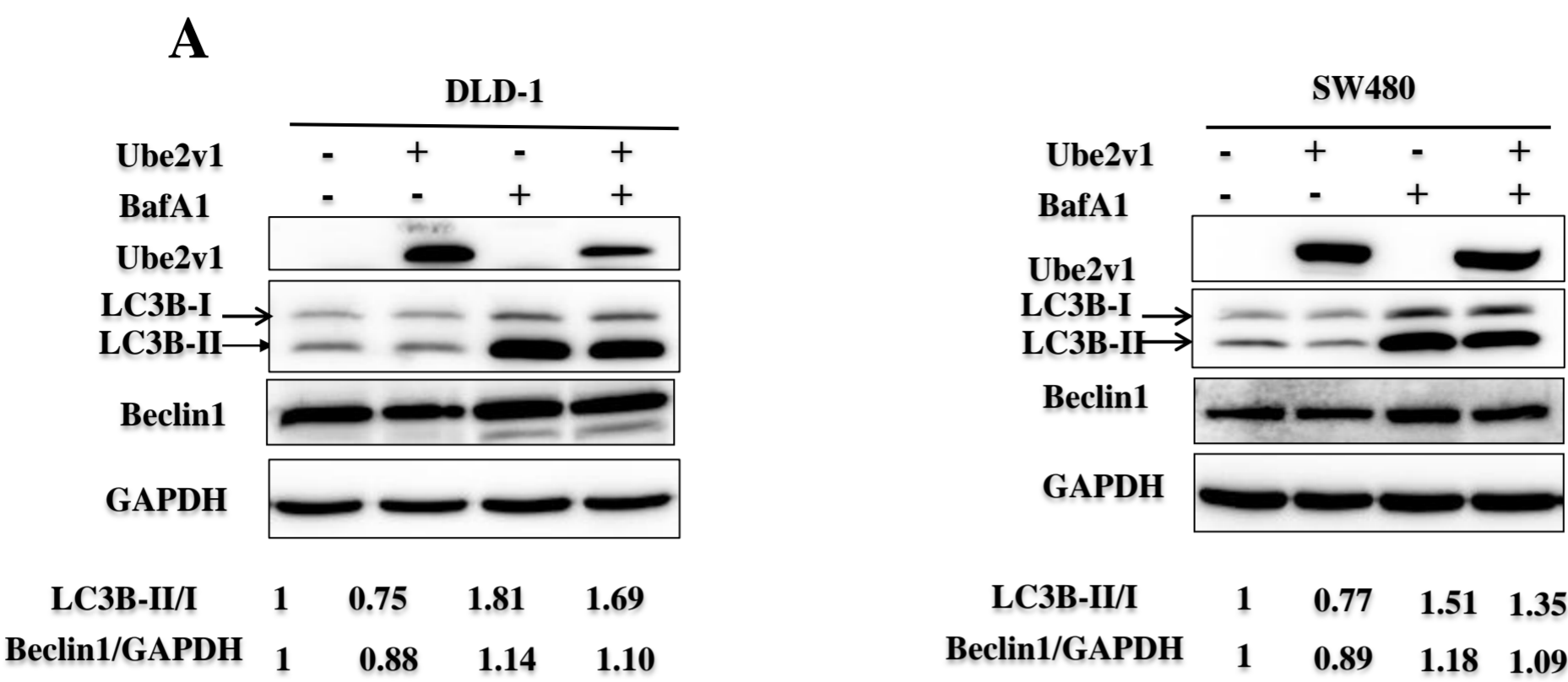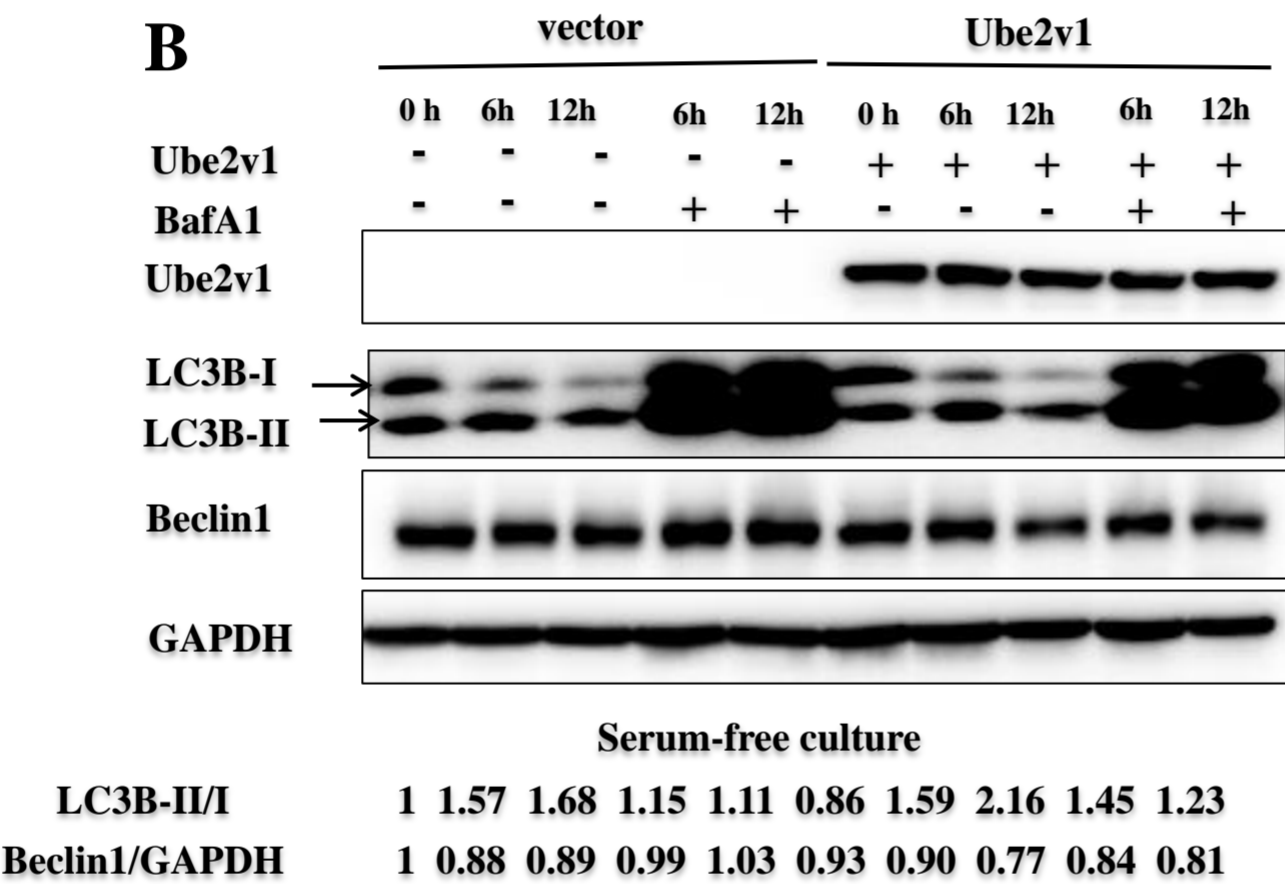

Figure S2

A

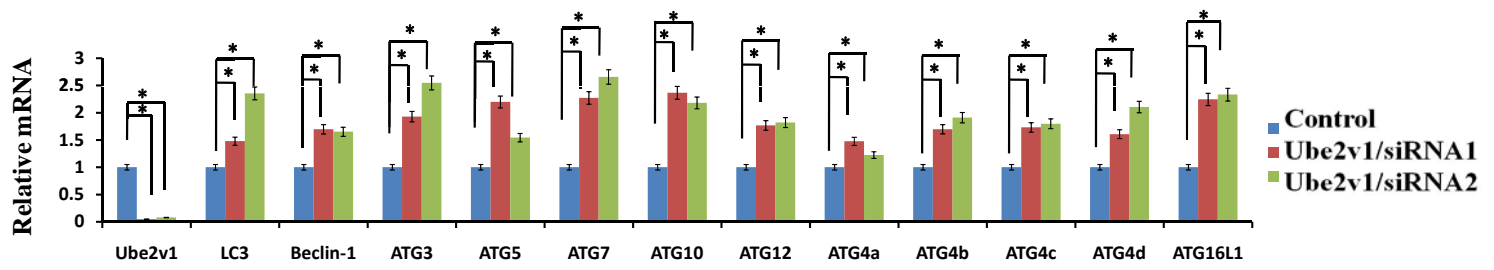

B

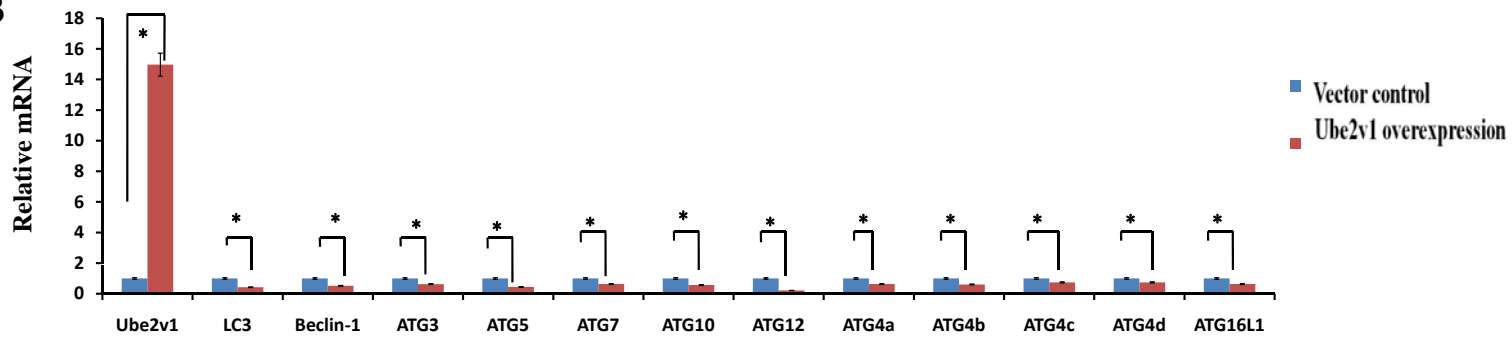

C

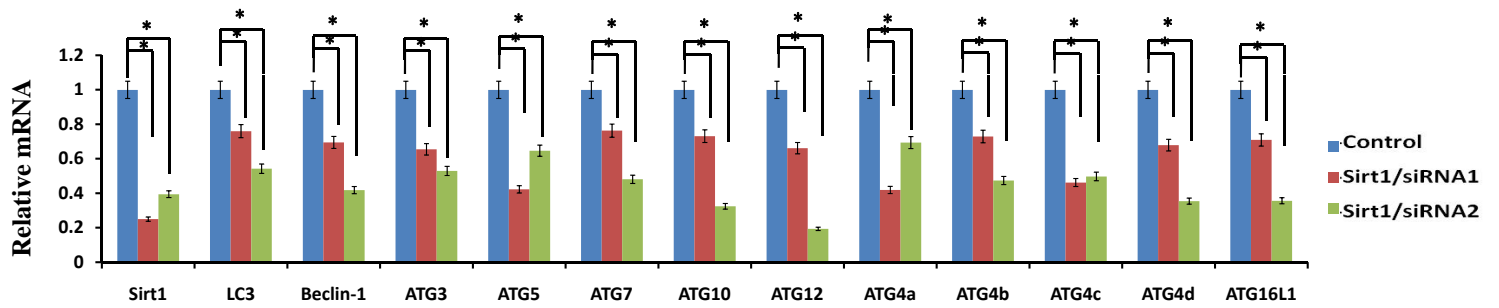

D

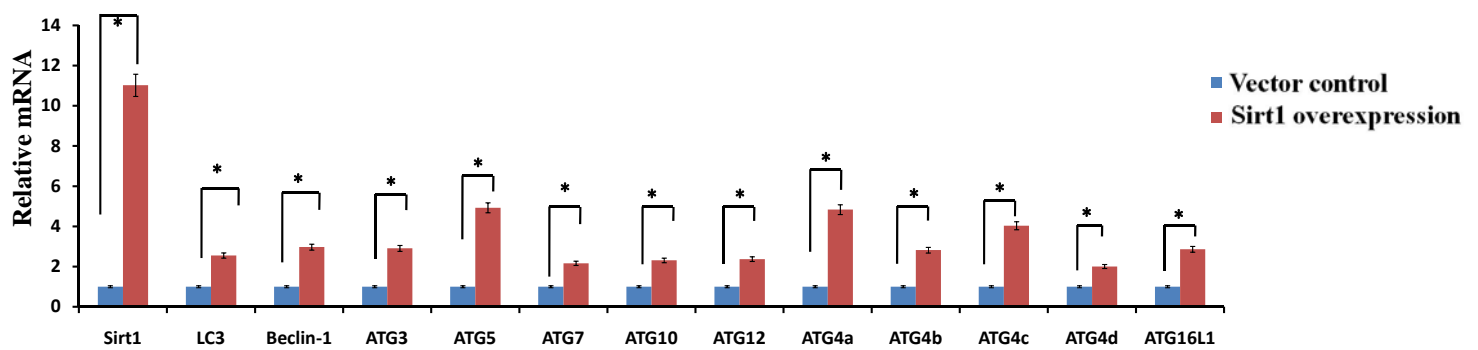

Figure S3

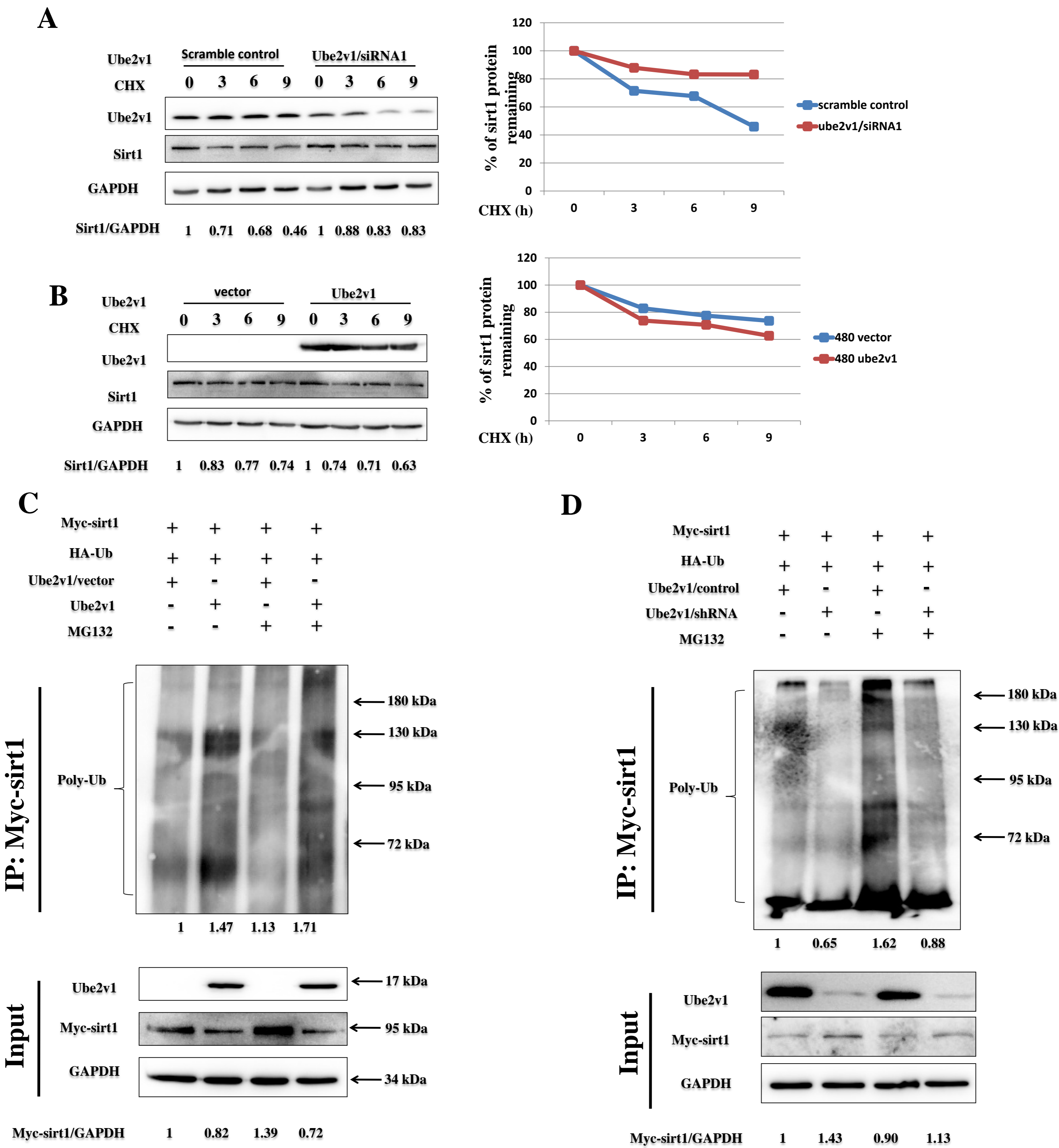

Figure S4

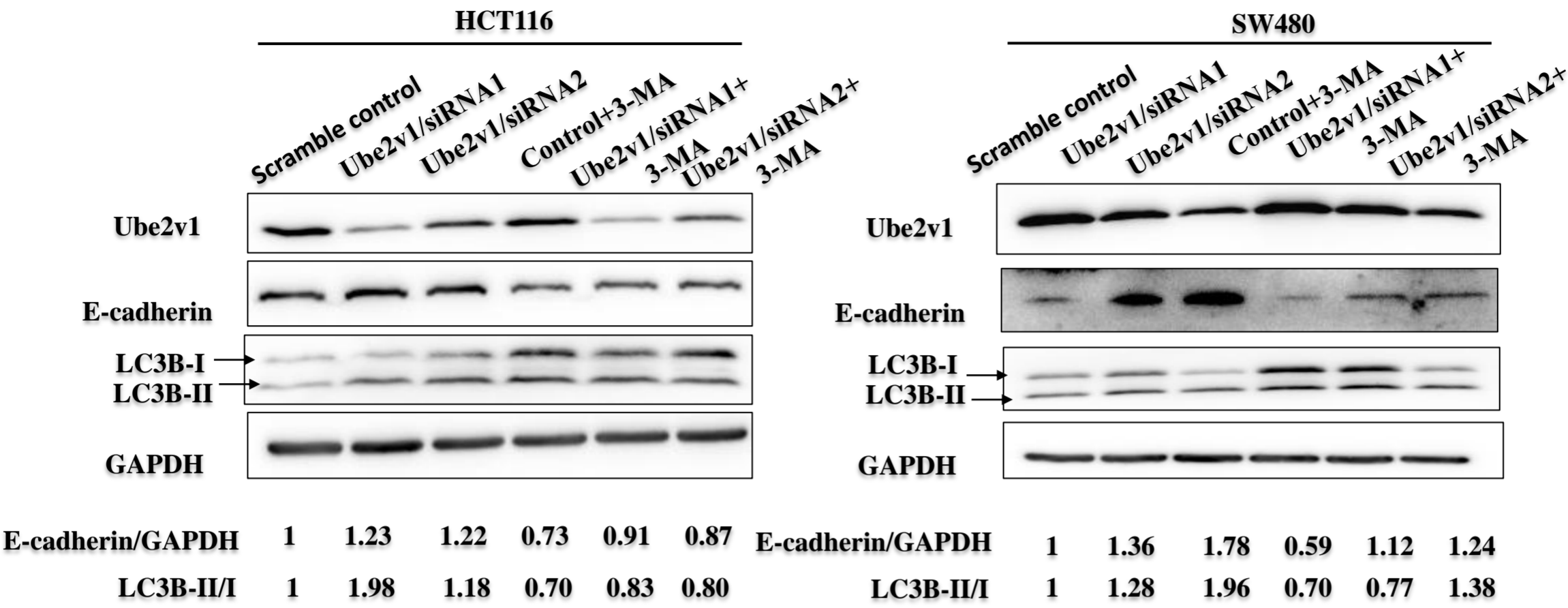

A

Figure S5

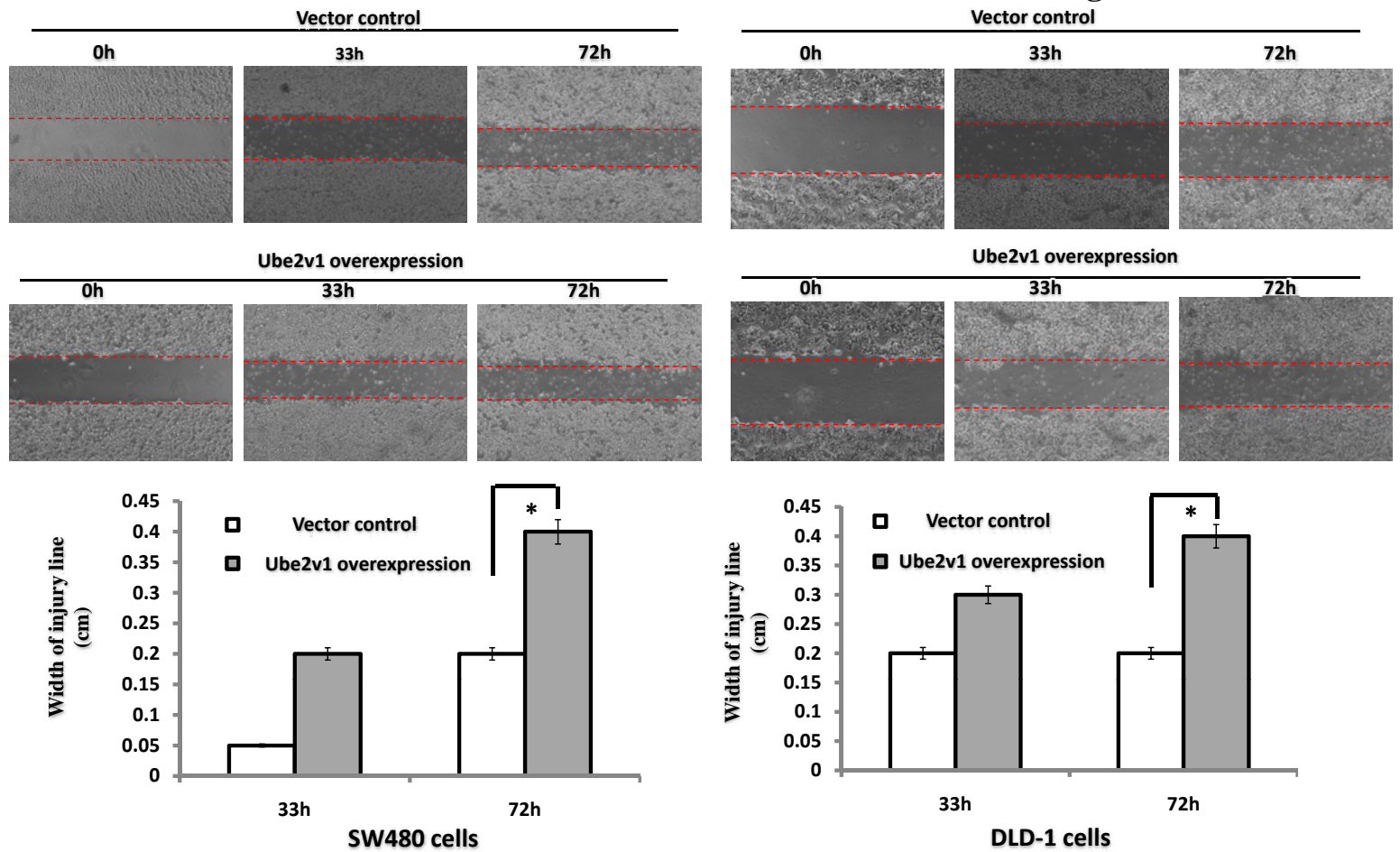

B

## Migration

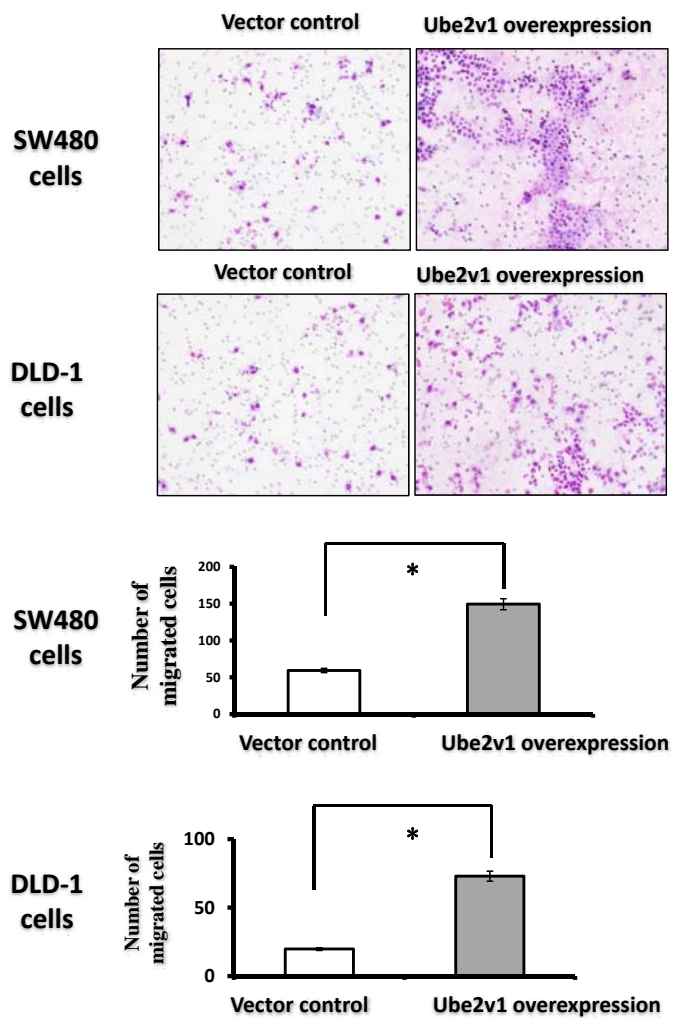

C

## Invasion

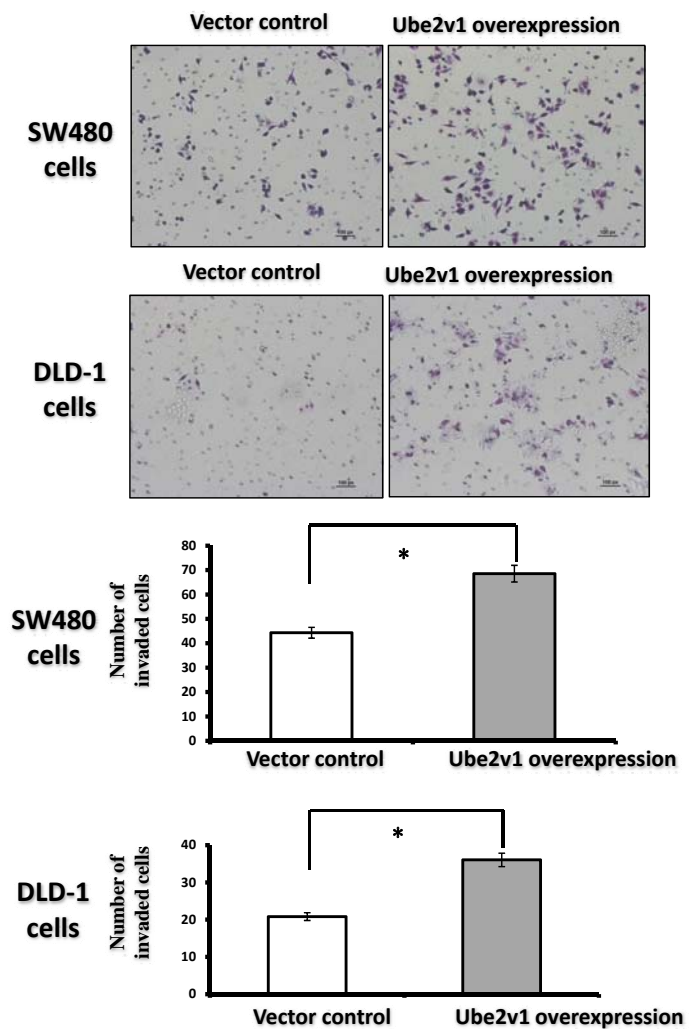

Figure S6

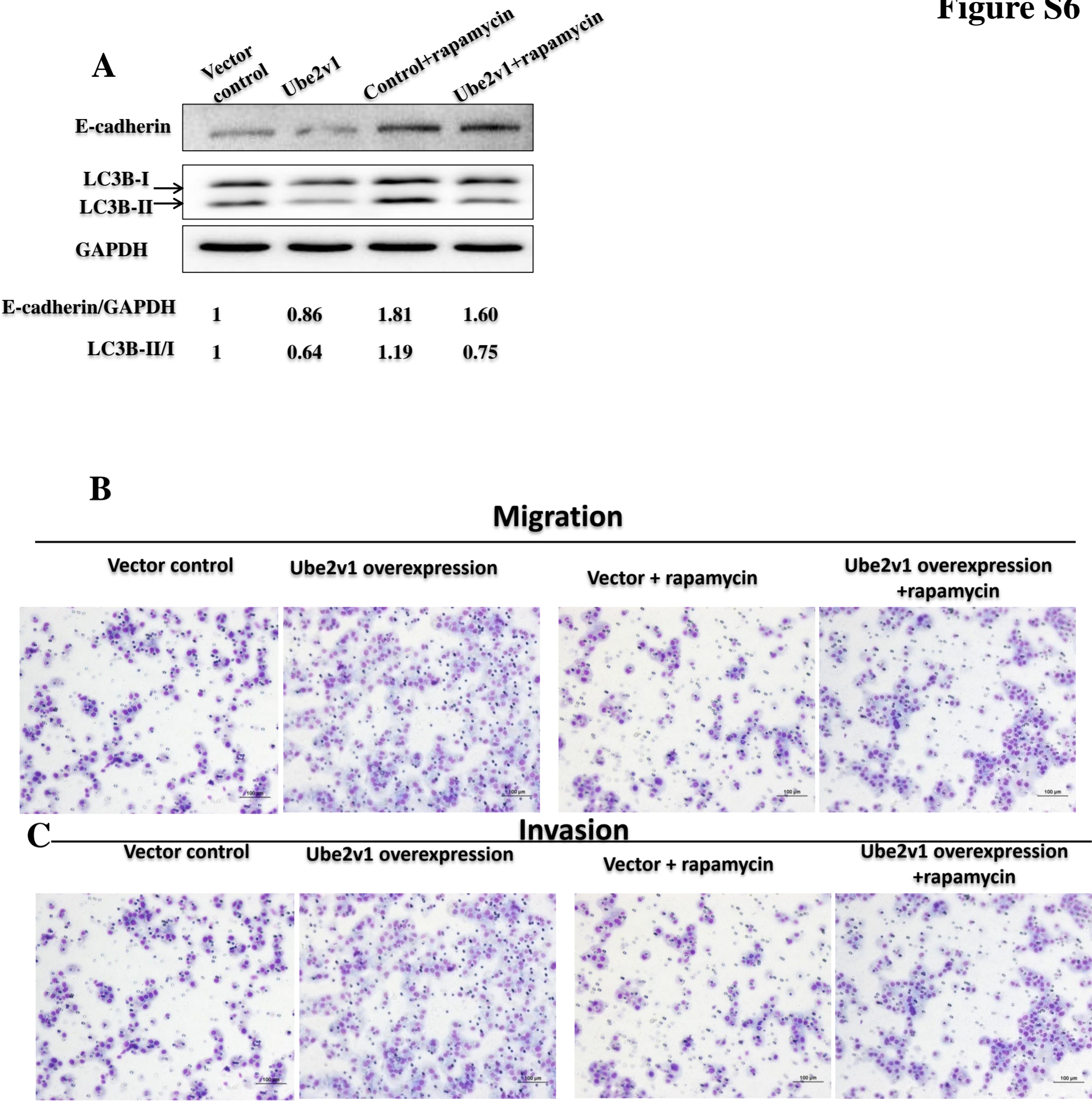

**Figure S7**

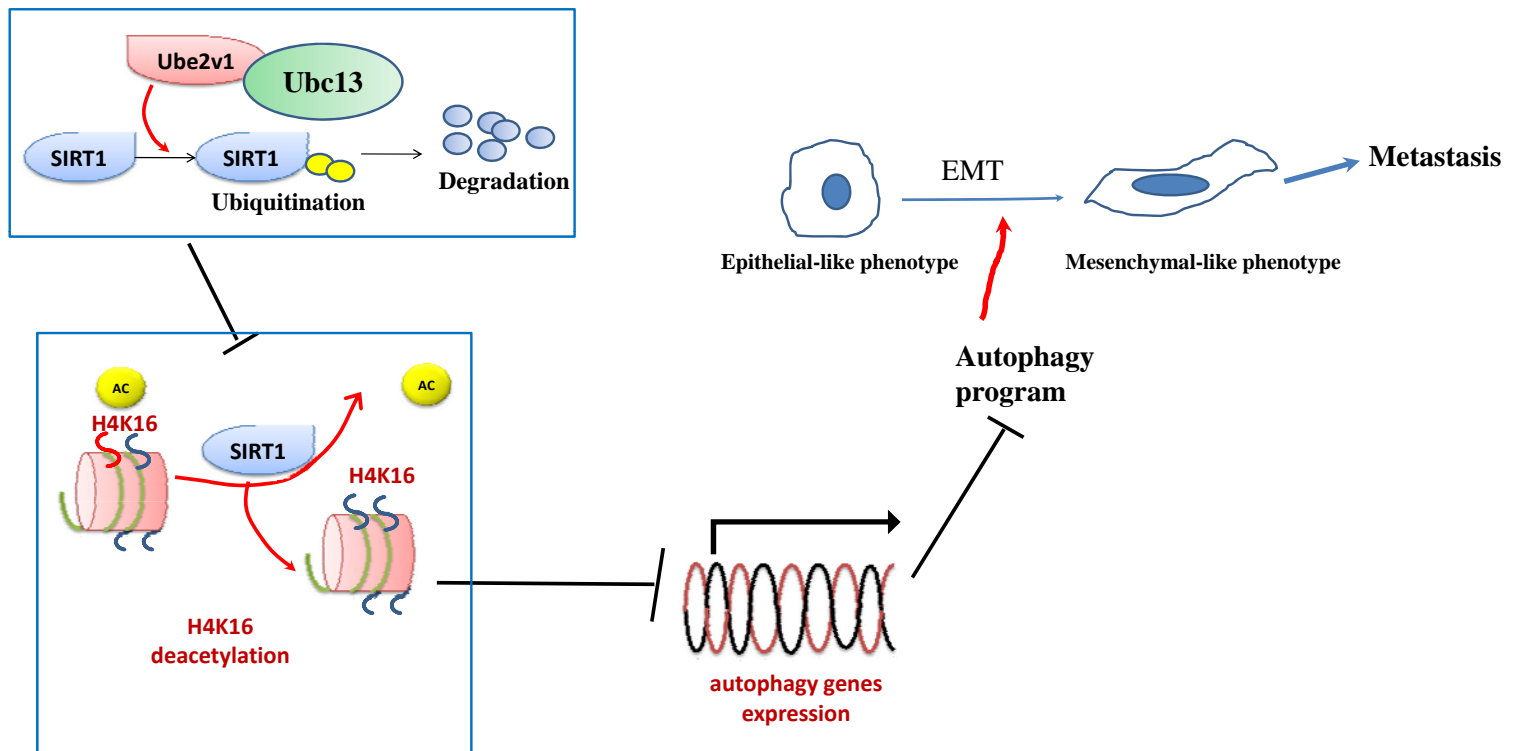

Supplement: Supplementary file 3 — Figure S1. Protein expressions of LC3-II and Beclin1 were examined by western blots when Ube2v1 was overexpressed in DLD-1 and SW480 cells under both normal medium culture condition (a) and starvation in Hank’s buffered saline solution (HBSS) (b). The cells were treated with Bafilomycin A1(BafA1), an autophagy inhibitor which blocks autophagosome–lysosome fusion. Figure S2. The effects of Ube2v1 on gene expressions of autophagy genes. A-B. mRNA levels of autophagy genes (LC3, Beclin1, ATG16L1, ATG3, ATG5, ATG7, ATG12, ATG10, ATG4a, ATG4b, ATG4c and ATG4d) were detected by qPCR analysis when Ube2v1 expression was knocked down (a) or overexpressed (b) in SW480 cells. C-D. mRNA levels of autophagy genes (LC3, Beclin1, ATG16L1, ATG3, ATG5, ATG7, ATG12, ATG10, ATG4a, ATG4b, ATG4c and ATG4d) were detected by qPCR analysis when Sirt1 expression was knocked down (c) or overexpressed (d) in SW480 cells. Figure S3. The effects of Ube2v1 on stabilization and ubiquitination of Sirt1 in CRC cells. The expression of Sirt1 was detected by western blotting in shRNA-transduced cells (a) or Ube2v1 overexpressed (b) SW480 cells treated with cyclohexamide (CHX) (100 μg/ml) for the indicated time intervals. The intensity of endogenous Sirt1 expression for each time point was quantified by densitometry. c Ubiquitination assays of exogenous Sirt1 in the lysates from SW480 cells cotransfected with GFP-Ube2v1, HA-Ub, Myc-Sirt1 or vector control. The cells were treated with or without MG132 (20 μM) before harvest and then immunoprecipitated them with anti-myc antibody. d Ubiquitination assays of exogenous Sirt1 in the lysates from SW480 cells cotransfected with HA-Ub, Myc-Sirt1 in the lysates from SW480 cells stably expressing Ube2v1 shRNA(shRNA/Ube2v1) or shRNA (shRNA/Control). Figure S4. Expressions of E-cadherin after Ube2v1 knockdown with stimulation of Autophagy inhibitor, 3-Methyladenine (3-MA) (5 mM) for 24 h. Figure S5. The effects of Ube2v1overexpression on wound-healing, migration [file 13045_2018_638_MOESM3_ESM.pdf]
